# In situ enzymatic generation of Au/Pt nanoparticles as an analytical photometric system: proof of concept determination of tyramine

**DOI:** 10.1007/s00604-023-05698-y

**Published:** 2023-03-06

**Authors:** Javier Camacho-Aguayo, Susana de Marcos, Carlos Felices, Javier Galbán

**Affiliations:** Nanosensors and Bioanalytical Systems (N&SB), Analytical Chemistry Department, Faculty of Sciences, Instituto de Nanociencia y Materiales de Aragón (INMA, Unizar-CSIC), University of Zaragoza, E50009, Zaragoza, Spain

**Keywords:** In situ generation, Enzymatic method, Bimetallic Au/Pt nanoparticles, Bimetallic Au/Pd nanoparticles, Tyramine, Tyramine oxidase, Photometric detection

## Abstract

In situ enzymatic generation of bimetallic nanoparticles, mainly Au/Pt, overcomes the drawbacks (continuous absorbance drift, modest LOQ, and long-time reaction) observed when AuNP alone are produced. In this study, Au/Pt nanoparticles have been characterized by EDS, XPS, and HRTEM images using the enzymatic determination of tyramine with tyramine oxidase (TAO) as a model. Under experimental conditions, the Au/Pt NPs show an absorption maximum at 580 nm which can be related to the concentration of tyramine in the range 1.0 × 10^-6^M to 2.5 × 10^-4^M with a RSD of 3.4% (*n* = 5, using 5 × 10^-6^M tyramine). The Au/Pt system enables low LOQ (1.0 × 10^−6^ M), high reduction of the absorbance drift, and a significant shortening of the reaction time (i.e., from 30 to 2 min for a [tyramine] = 1 × 10^−4^M); additionally, a better selectivity is also obtained. The method has been applied to tyramine determination in cured cheese and no significant differences were obtained compared to a reference method (HRP:TMB). The effect of Pt(II) seems to involve the previous reduction of Au(III) to Au(I) and NP generation from this oxidation state. Finally, a three-step (nucleation-growth-aggregation) kinetic model for the generation of NPs is proposed; this has enabled us to obtain a mathematical equation which explains the experimentally observed variation of the absorbance with time.

**Graphical abstract:**

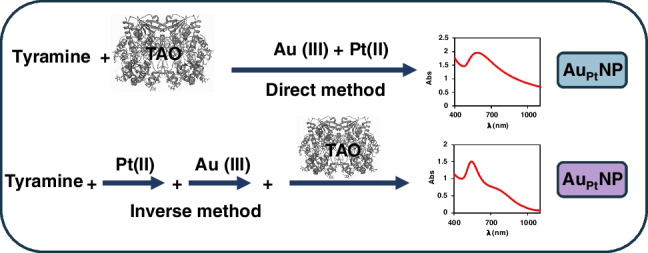

**Supplementary Information:**

The online version contains supplementary material available at 10.1007/s00604-023-05698-y.

## Introduction

Spectrophotometric enzymatic methods of analysis constitute one of the most useful and widespread alternatives for the determination of many different families of analytes. Many of these methods are based on an oxidase-type catalyzed reaction (main reaction) where O_2_ is oxidized to H_2_O_2_. Such methods require coupling an indicating reaction involving horseradish peroxidase (HRP) and a chromogen (R) such as ABTS or TMB [1] which are oxidized by H_2_O_2_.

1 $${\mathrm{H}}_2{\mathrm{O}}_2+{\mathrm{nR}}_{\mathrm{red}}\ \overset{\ \mathrm{HRP}\ }{\to }\ {\mathrm{H}}_2\mathrm{O}+{\mathrm{nR}}_{\mathrm{ox}}$$

Although they give good results, they also have several drawbacks [2, 3]. For example, (i) *R*_ox_ can react with the amino acids of the main reaction and HRP; (ii) *R*_red_ can react with the product of the main reaction or other chemicals present in the sample; (iii) *R*_ox_ is unstable (it suffers disproportionation). Also, HRP presents additional lateral reactions with the analyte or the product of the oxidase reaction [4].

Nanomaterials offer new possibilities for the indicating reaction (1), acting in two ways: as HRP mimics and as a chromophore/chromogen. Regarding HRP mimics, many nanomaterials (iron-based or carbon-based) [5] have been described which present sufficient catalytic activity to replace HRP in the reaction (1).

As a chromophore or chromogen, some nanomaterials allow alternative indicating mechanisms different from (1), the most important being [6, 7] (i) nanomaterial modification. The spectroscopic properties of nanomaterials (surface plasmon resonance band (SPR)) change (absorbance or wavelength) as a consequence of the enzymatic reaction, (ii) nanomaterial generation. In this case, nanomaterials are generated from the corresponding ions as a consequence of the enzymatic reaction. Papers by Pavlov [6] describe quantum dots (QD) generation where during the enzymatic reaction the analyte is hydrolyzed to sulfide which reacts with Cd(II), previously added to the medium, to form QD.

In a previous paper [8], we presented an alternative indicating method based on the formation of AuNP from Au(III) during tyramine (Tyr) enzymatic oxidation to the aldehyde form (Tyr_ald_) catalyzed by tyramine oxidase (TAO).

2 $$\mathrm{Tyr}+{\mathrm{O}}_2\overset{\ \mathrm{TAO}\ }{\to }{\mathrm{Tyr}}_{\mathrm{ald}}+{\mathrm{H}}_2{\mathrm{O}}_2$$

The analytical signal was the appearance of the SPR band which was measured. The mechanism was not fully clarified but we concluded that it is a combination of two processes:

1. Au(III) reduction by the product of the enzymatic reaction:

3 $$\mathrm{Au}\left(\mathrm{III}\right)+{\mathrm{Tyr}}_{\mathrm{ald}}\rightleftarrows {\mathrm{Au}}^0+{\mathrm{Tyr}}_{\mathrm{acid}}$$

2) Au(III) acting as O_2_ regenerating the active center of the enzyme

4 $$\mathrm{Au}\left(\mathrm{III}\right)+{\mathrm{TAO}}_{\mathrm{red}}\rightleftarrows {\mathrm{TAO}}_{\mathrm{ox}}+{\mathrm{Au}}^0$$

This methodology allowed us to determine tyramine. Despite the advantages of the method, some aspects remained unresolved: (i) low sensitivity, as the method allows the analyte determination with a quantification limit of 2.5 × 10^−5^M which is much higher than the classical colorimetric; (ii) interference from other biogenic amines present in the samples; (iii) the method rate which requires working at a high temperature; and (iv) the fact that after the reaction a long continuous signal drift is observed which affects the method reproducibility and complicates the appropriate choice of measurement time. In this paper, we have tried to overcome some of these problems replacing Au(III) by another metal ion able to produce nanoparticles (or mixtures of Au(III) with other metal ions). When nanoparticles are formed from Au(III) plus Pt(II) (or Pd(II)), some of the above-mentioned drawbacks disappear, especially the lower sensitivity, the necessity of working at high temperatures, and the long absorbance drift, improving the analytical possibilities of this methodology.

## Experimental section

### Reagents and solutions

Tyramine oxidase (TAO) (EC 1.4.3.6) with an activity of 4.6 U × mg^−1^ was obtained from Sekisui Diagnostics (T-25). Na_2_HPO_4_ (S9763) and Na_2_PO_4_ (S9638) for the buffer solutions, gold chloride hydrate solid (254169), and potassium tetrachloroplatinate (206075) (which were dissolved in MiliQ water to obtain a 50-mM solution), all biogenic amines (tyramine (T2879), putrescine (P7505), cadaverine (C8561), and histamine(53300)), other enzymes and proteins (catalase (C40), laccase (40452), glucose oxidase (GOx) (9001-37-0), and bovine serum albumin (BSA)(A7906)) and the hydrogen peroxide (7722-84-1) solution were obtained from Sigma Aldrich.

The product of the reaction (Tyr_ald_ = p-hydroxybenzaldehyde) was synthesized using the enzymatic reaction (2) and a constant supply of oxygen; catalase was also added to eliminate the H_2_O_2_ (7722-84-1) generated during the reaction. Finally, the solution was ultra-centrifuged in order to eliminate the TAO and catalase employed.

### Apparatus

A Tecnai F30H–7650 microscope (scanning and transmission mode, STEM) (FEI, The Netherlands, https:// www.fei.com) and an XPS Spectrometer Kratos AXIS Supra equipped with an Al Kα (120W) X-ray source were used for morphology and composition characterization of the nanoparticles. Purification and isolation of different samples were carried out using a Koch centrifuge from Bunsen and Amicon-Ultra 10kDa centrifugal filters from Milipore. UV-vis molecular absorption measurements were performed on a Specord 210 Plus spectrophotometer (from Jena) and an Agilent 8453 diode array spectrophotometer. One-centimeter cuvettes were used in all cases. The Millipore MiliQ H_2_O system was used for water purification.

### Sample treatment

About 10 g of cured cheese were weighed (with ± 0.01 precision), leached with 30 mL 5% trichloroacetic acid for 30 min. Then, the mixture was centrifugated (20 min, 4 °C, 5000 rpm), the solid phase was discarded, and the supernatant solution was neutralized with NaOH (2M). Next, a second centrifugation was done in the same conditions, and the supernatant was double filtered: firstly, through a 25-mm diameter nylon membrane filter (ALBET-NY-045-25-BL) and, secondly, through a 10-kDa centrifugal filter. Finally, the solution was adjusted to 50 mL with 0.1 M pH 7 phosphate buffer.

### Measurement procedure

One thousand nine hundred forty μL of TAO (0.5U/mL) dissolved in the phosphate buffer solution (0.1 M) and 20 μL of the corresponding standard solution or sample were added to the cuvette under stirring. After waiting 5 min, 40 μL of 50 mM HAuCl_4_ and 80 μL of 50 mM K_2_PtCl_4_ were added to the cuvette. The formation of the nanoparticles was followed by measuring the variation of absorbance at 580 nm.

## Results and discussion

### Ion metals used for nanoparticles generation: bimetallic Au_Pd_ nanoparticles

**Fig. 1:**
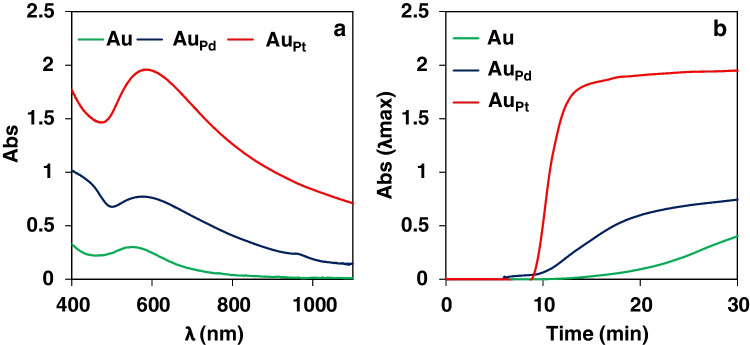
**a**) Absorption spectra and **b**) absorbance versus time representations (*λ*_max_), obtained during the enzymatic reaction of tyramine (10^−4^M) in phosphate buffer pH = 7: green line indicates the following: AuNP ([Au(III)] = 1 mM, [TAO] = 0.5 U/mL, 40 °C, 0.1M buffer). Blue line indicates the following: Au_Pd_NP ([Au(III)] = 0.5 mM, [Pd(II)] = 1.5 mM, [TAO] = 0.25 U/mL, 40 °C, 0.3M buffer). Red line indicates the following: Au_Pt_NP ([Au(III)] = 0.5 mM, [Pt(II)] = 1.0 mM, [TAO] = 0.125 U/mL, 25°C, 0.1M buffer)

To solve the problems associated with AuNP generation as the indicating reaction, NP generation was firstly assayed using other metals able to give absorption bands in the UV-vis region due to either plasmon resonance or inter-band transitions [9]. The following ions were tested: Ag(I), Cu(II), Ir(III), Rh(IV), Pt(II), and Pd(II), and the same enzymatic reaction was used for a better evaluation of the analytical improvements. Some of them (such as Pt(II) or Pd(II)) were partially reduced to metal but, except with Cu(II), no NP formation was observed during the enzymatic reaction. However, using solutions containing mixtures of either Au(IIII)/Pd(II) or Au(III)/Pt(II), positive results were observed. These new NP (Au_Pd_ and Au_Pt_, respectively) showed slight changes in the wavelength of the absorption spectra (Fig. 1a) but, more significantly, in the sensitivity and the kinetic of the reaction (Fig. 1b) compared to the use of Au(III) alone .

**Fig. 2:**
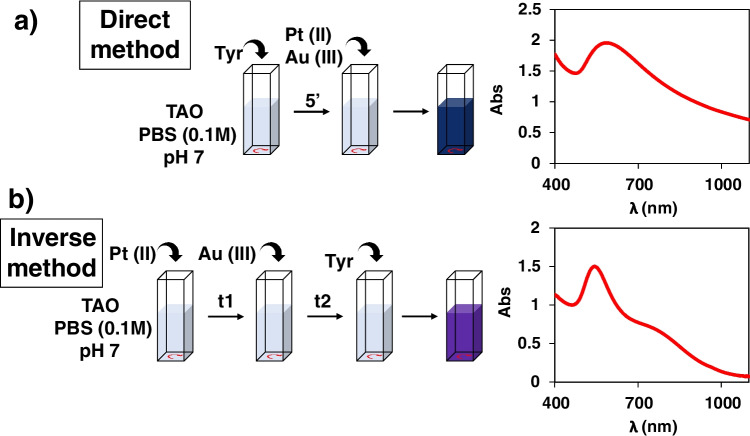
Schematic representation of the **a**) direct and **b**) inverse method, and the final molecular absorption spectra obtained in each case. Experimental conditions for the direct method as indicated in Fig. 1; for the inverse method ([Au(III)] = 0.5 mM, [Pt(II)] = 0.5mM, [TAO] = 0.50 U/mL, 40 °C, 0.1M phosphate buffer pH = 7)

The Au_Pd_ system was first studied using the so-called Direct method (see below in Fig. 2). The best experimental conditions for the Au(III) (Fig. S1), Pd(II) (Fig. S2), TAO (Fig. S3), pH, and buffer concentration (Fig. S4) were chosen using as the optimization criteria the absorbance at the maximum measurement time (usually 60 min, Abs_60_), the minimum reaction time, the minimum drift of the absorbance versus time (Abs = f(t)) representations, and the minimum widening of the spectra. In the best conditions found, bimetallic NP of different shapes were formed (as was demonstrated by the XPS and HRTEM studies (Fig S5)), and the absorbance at 550 nm was measured after 20 min of reaction. The analytical figures of merit slightly improved those obtained using Au(III): lower absorbance drift and reaction time, higher molar absorptivity of the NP formed but similar linear response range for tyramine (from 5 × 10^−5^ M to 2.5 × 10^−3^ M) (Fig. S6) and precision (2.3% RSD, *n* = 5, 0.25mM Tyramine); moreover, the interference of histamine was directly proportional to its concentration (Fig. S7). These results allowed some of the objectives to be achieved (decreasing the signal drift and a better handling of the interference of histamine), but better results were obtained using Au_Pt_.

### Bimetallic Au_Pt_ nanoparticles: Direct and Inverse methods

Au(III) and Pt(II) present a very rich and complex reactivity with each other. They are initially prepared as chloro-complexes (AuCl_4_^−^ and PtCl_4_^2−^ respectively); in this chemical form, Pt(II) is able to reduce Au(III) [10, 11] according to:

5 $$\mathrm{Au}\left(\mathrm{I}\mathrm{I}\mathrm{I}\right)+\mathrm{Pt}\left(\mathrm{I}\mathrm{I}\right)\rightleftarrows \mathrm{Au}\left(\mathrm{I}\right)+\mathrm{Pt}\left(\mathrm{I}\mathrm{V}\right)$$

However, depending on the chloride concentration, Au(III) and Pt(II) can form different aquo-chloro-complexes (Au(H_2_O)_x_Cl_6-x_ and Pt(H_2_O)_y_Cl_4-y_) which affect their redox capability in such a way that reaction (5) could not occur, or Au(III) could even be reduced to Au^0^. Moreover, both Au(III) and Pt(II) can form complexes with biogenic amines [12] and with anions present in buffer solutions, especially phosphate which is necessary to stabilize AuNP [13], but the rates at which these complexes are formed (i.e., the corresponding kinetic constants) have not been clearly established. Then, the predominant chemical species of gold, platinum and analyte, at the beginning of the reaction, will depend on the order of addition of the different reagents and the time elapsed between these additions; consequently, the final result obtained will also be different. Taking this into account, two working methods were finally tested: the Direct and the Inverse (Fig. 2).

In the Direct method, the enzymatic reaction between tyramine and TAO in the appropriate buffer is first carried out (during a certain time, *t*_1_) in the spectrophotometer cuvette. After that, Au(III) and Pt(II) are simultaneously added, which means that reactions (2) and (5) are produced simultaneously. This method is similar to that previously used [10], but without the simultaneous addition of Pt(II). In the Inverse method, first Pt(II), then Au(III) (after a time, *t*_1_), and finally tyramine (after a time *t*_2_) are added to the spectrophotometer cell containing TAO in buffered media, indicating that (5) can be carried out previously to the enzymatic reaction.

Taking this into account, it is expected that the characteristics of the NP formed would be different in both cases. Figure S8 shows the TEM, EDX, and XPS analysis corresponding to those formed by the Direct method. According to the XPS, they are mainly composed of Au^0^ and a small amount of Pt^0^. The TEM shows strong aggregation which prevents the accurate determination of their size and shape but confirms the NP formation and explains the increase of the baseline. Figure S9 shows the results corresponding to the Inverse method. Besides Au^0^, the XPS shows two shoulders close to the maximum Au4f (i.e., the doublet 4f_5/2_, 4f_7/2_) which are associated with gold oxides; moreover, the signals observed for Pt are mainly associated with an oxidized platinum species. The morphology shows smaller aggregation and that nanoparticles are associated in trios. Finally, Fig. 2 shows the characteristic molecular absorption spectra of both types of nanoparticles. Those corresponding to the Direct method show a maximum at 550 nm while those of the inverse method show maxima at 550 nm and 800 nm; the latter spectra are consistent with those previously reported by Zohar et al. [14], being due to the trios of nanoparticles. In order to detect the presence of absorption bands in the NIR, the spectra were extended up to 1500 nm but no new peaks were detected.

### Choosing the appropriate method

The Direct and Inverse methods were experimentally studied in parallel. With the Inverse method, several optimization studies were carried out:

A) Order of addition of metal ions

When Au(III) was added first, the kinetic of the reaction was slower and the signal showed a severe drift (similar to that observed when Pt(II) was not added) (Figure S10), but when Pt(II) was added first, the drift nearly disappeared and good results were obtained. It is important to realize that when Au(III) is added first, Au(III)/phosphate is already formed when Pt(II) is added, so Pt(II) does not seem able to reduce Au(III) when it has been previously complexed by phosphate.

B) Reaction times (*t*_1_ and *t*_2_) optimization

Figure S11 shows that the kinetic of the reaction was almost independent of *t*_1_ and *t*_2_. The final absorbance slightly increased with these times and the shape of the spectra was not affected by *t*_2_ but changed with *t*_1_ (the lower the *t*_1,_ the higher the absorbance at 540 nm and the lower the absorbance at 830 nm).

III) Ion metal concentrations and ratio

Different ion metal concentrations and proportions were studied. It was found that a high concentration causes the system to precipitate and an increase in the gold concentration causes a high drift of the signal without achieving stabilization (Figure S12). This seems to indicate that free Au(III) is responsible for the signal drift and that this does not occur when Au(I) is used.

IV) Tyramine concentration

**Fig. 3:**
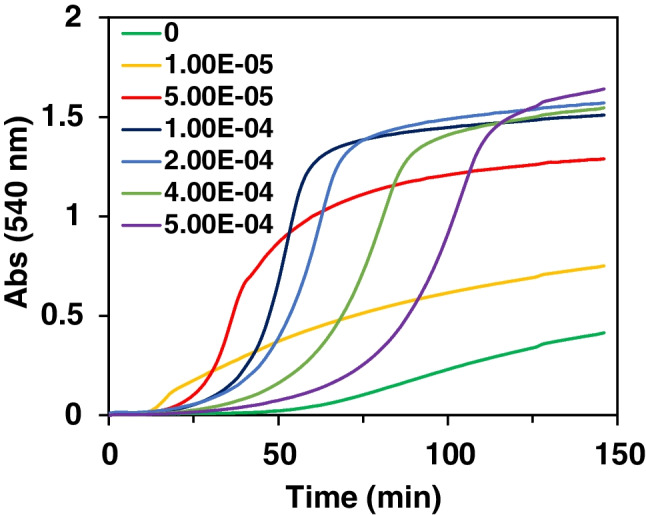
Inverse method: variation of the absorbance at *λ* = 540 nm with the reaction time for different concentrations of tyramine. [Au (III)] = 0.5 mM; [Pt (II)] = 0.5 mM; [TAO] = 0.5 U/mL; phosphate buffer 0.1 M pH 7; 40 °C

The most relevant results were obtained during the calibration study. Figure 3 corresponds to Abs = f(t) representations obtained at 540 nm (similar results were obtained at 830 nm; see Figure S13) and clearly shows that the kinetic of the NP formation is in inverse proportion to the tyramine concentration; this behavior is just the opposite to that observed in the Direct method (see below).

Two explanations have been considered:

1. According to (4), ionic gold (Au(I) in this case) can regenerate the active center of the enzyme in competition with O_2_. Although O_2_ is preferred, when the concentration of tyramine is high, the remaining O_2_ concentration will be very low, so the regeneration by Au(I) will be favored and Au^0^ will be formed around the active center of the enzyme. This distorts the geometry of the active center, making the reaction kinetic slower. Therefore, the higher the tyramine concentration, the higher the amount of Au^0^ formed and the lower the kinetic of the reaction.
2. It has been reported in the literature that AuNP can catalyze the disproportionation of H_2_O_2_ similarly to catalase, partially regenerating the O_2_ consumed, but this catalytic effect decreases as the AuNP concentration increases [15], namely as the tyramine concentration increases. To test this hypothesis, several assays have been performed using catalase which suppresses the H_2_O_2_ formed_._ No changes were observed in any case (Figure S14), so this mechanism can be discarded.

From these results, it can be concluded that Au(I) presumably acts as an enzyme cofactor in the Inverse method.

E) Analytical figures of merit

Different parameters can be used to determine tyramine by this method. The area of the Abs = f(t) representation (during an interval of time) gives a short linear range. Finally, the absorbance at 540 nm and 830 nm (at 120 min) was finally proposed. Figure S15 shows that the Abs_540_-Abs_830_ nm follows a second order polynomial equation:

6 $${\left({\mathrm{Abs}}_{540}-{\mathrm{Abs}}_{830}\right)}_{120\ \min }=-22\ {\left[\mathrm{Tyramine}\right]}^2+-8.8\ \left[\mathrm{Tyramine}\right]+0.057$$

for a tyramine concentration range between 0.01 mM (LoQ) up to 0.2 mM, with a 5% RSD (1.0 × 10^−4^M, *n* = 5).

### Direct method

A) Optimization of analytical conditions

The effect of the Pt(II) and Au(III) concentrations were first considered. The enzymatic reaction was tested using 4x4 matrix Pt(II)xAu(III) concentrations. The results obtained are detailed in Figure S16. For any Pt(II) concentration (Figure S16A), the higher the Au(III) concentration, the faster the reaction and the higher the final absorbance, but the flatter the plasmon band. For any Au(III) concentration (Figure S16B), the higher the Pt(II) concentration, the higher the signal, but no appreciable changes were observed in the kinetic of the reaction. Finally, 0.5 mM Au(III) and 2 mM Pt(II) were chosen as optimum considering the kinetic of the reaction and the shape of the plasmon band (Figure S16C).

As previously reported, a phosphate buffer is the most suitable for nanoparticle formation using this enzymatic reaction. Since the enzymatic reaction only occurs in the middle zone, the effect of the pH was studied in the 6–8 range. As shown in Figure S17, even in this narrow range, the pH greatly affects the shape of the spectra and the kinetic of the reaction. A pH of 7 was considered as a good compromise solution.

The effect of the enzyme concentration (TAO) is shown in S18, and the results indicate that an optimum concentration (0.125 U/mL) is obtained from which the kinetic of the reaction becomes slower and the absorbance at 60 min (maximum measurement time, Abs_60_) decreases. We have previously observed this behavior in determinations based on the enzymatic generation of nanomaterials [8, 16], and it is accounted for considering that the enzyme concentration favors the nucleation step (Au^0^ seeds), but it goes against the growth step (Au^0^ aggregation to give NP).

The temperature was studied, and it was observed that an increase in temperature favors the nanoparticle formation. However, this also produces an increase in the blank signal (Figure S19).

B) Initial considerations of the mechanism

Prior to the elucidation of the kinetic model, it was first necessary to consider the possible mechanisms of nanoparticle formation. For this reason, the reducing capabilities of tyramine, the enzyme, the product of the reaction, and the peroxide were considered. It is important to highlight that unlike in previous studies where the product of the reaction was not able to fully explain the formation of the nanoparticles, it seems that in this new approach the product plays a much more important role, being responsible for the NPs formation. It is important also to highlight the stabilizing role of the enzyme (TAO), since no similar results were obtained with other enzymes and proteins (Fig S20). Moreover, the role of H_2_O_2_ was studied given that some earlier studies have indicated that it is able to grow previously formed NP, and other studies highlight its etching role. In this approach, if the experiment is carried out in the presence of catalase, which allows removing the H_2_O_2_ formed during the enzymatic reaction, the kinetic is affected, making the reaction slower. Otherwise, the more peroxide, the faster the nanoparticle formation. Nevertheless, peroxide is not able to generate NPs by itself (Fig S21). With all this information a kinetic model can be proposed.

III) Kinetic model of the NPs formation (tyramine concentration effect)

Previously [10], we derived a kinetic model (based on that of Watzky and Finke [17]) to explain the gold nanoparticles (AuNP) formation during the enzymatic reaction. This model involved a classical two-step mechanism (nucleation and growth) [18, 19]. A mathematical equation was also derived which described the S-shape behavior of the first-half of the Abs=f(t) (Fig. 1b, *t* < 30 min):

7 $$\mathrm{Abs}={\mathrm{K}}_{\mathrm{A}\mathrm{u}}\left(\frac{1-{\mathrm{e}}^{-{\mathrm{A}}_{\mathrm{A}\mathrm{u}}\mathrm{t}}}{{\mathrm{e}}^{-{\mathrm{A}}_{\mathrm{A}\mathrm{u}}\mathrm{t}}+{\mathrm{B}}_{\mathrm{A}\mathrm{u}}}\right)$$

being

8 $${\mathrm{A}}_{\mathrm{A}\mathrm{u}}={\mathrm{C}}_{\mathrm{A}\mathrm{u}}\left({\mathrm{k}}_{1,\mathrm{Au}}+\left(\frac{{\mathrm{k}}_{2,\mathrm{Au}}}{n_{\mathrm{A}\mathrm{u}}}\right){\mathrm{C}}_{\mathrm{Tyr}}\right)$$

9 $${\mathrm{B}}_{\mathrm{Au}}=\frac{{\mathrm{k}}_{1,\mathrm{Au}}}{\left(\frac{{\mathrm{k}}_{2,\mathrm{Au}}}{n_{\mathrm{Au}}}\right){\mathrm{C}}_{\mathrm{Au}}}$$

10 $${\mathrm{K}}_{\mathrm{Au}}=\left(\frac{\upvarepsilon_{\mathrm{Au}\mathrm{NP}}}{n_{\mathrm{Au}}}\right)\frac{{\mathrm{k}}_{1,\mathrm{Au}}}{\left(\frac{{\mathrm{k}}_{2,\mathrm{Au}}}{n_{\mathrm{Au}}}\right)}$$

In these equations *ε*_*AuNP*_ is the molar absorptivity of the AuNP formed, n_Au_ the number of atoms in each AuNP, C_Au_, and C_Tyr_ are the total Au(III) and tyramine concentrations, and k_1,Au_ and k_2,Au_ the kinetic constants of the nucleation and growth steps. However, the A_Au_ values derived from the experimental data were not able to fully explain the model because they randomly changed with the tyramine concentration.

In this paper, we have improved and extended the kinetic model to include the whole Abs = f(t) representation. To do so, in addition to the nucleation and growth steps given by:

11 $$\mathrm{Nucleation}:\mathrm{Au}\left(\mathrm{I}\right)+\mathrm{Tyr}\ \overset{\ \mathrm{k}_1\ }{\to }\ {\mathrm{Au}}^0$$

12 $$\mathrm{Growth}:\mathrm{Au}\left(\mathrm{I}\right)+\mathrm{Tyr}+{\mathrm{Au}}^0\ \overset{\ \mathrm{k}_2\ }{\to }\ \mathrm{AuNP}$$

(bear in mind that the NP formation is preceded by reaction (5)), the TEM image given in Fig. S8 was also considered. This figure shows that NPs tend to form aggregates which do not actually produce larger NP but merely a contact between their outer spheres; this process will distort the initial S-shape of the Abs = f(t) representation (due to a change in the molar absorptivity). Since the formation of aggregates does not require a chemical reaction, it should follow the Avrami equation for change of phase [20], which can be expressed as

13 $$\mathrm{Abs}={\mathrm{Abs}}_{\infty }\ \left(1-{\mathrm{e}}^{-{\mathrm{k}}_3{\mathrm{t}}^n}\right)$$

**Fig. 4:**
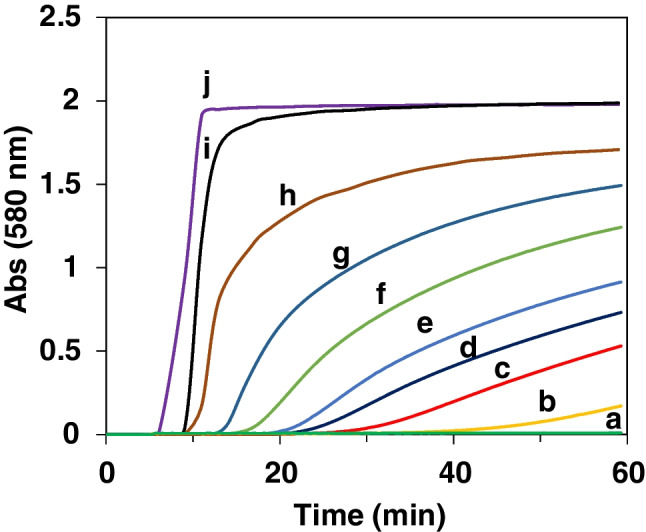
Direct method: Abs = f(t) representations at *λ* = 580 nm for different concentrations of tyramine (M). [TAO] = 0.125 U/mL; [Au (III)] = 0.5 mM; [Pt (II)] = 1 mM; phosphate buffer 0.1 M pH 7; 25 °C. **a** blank; **b** 2.5 × 10^−6^M; **c** 5.0 × 10^−6^M; **d** 7.5 × 10^−6^M; **e** 1.0 × 10^−5^M; **f** 1.5 × 10^−5^M; **g** 2.5 × 10^−5^M; **h** 5.0 × 10^−5^M; **i** 1.0 × 10^−4^M; **j** 2.5 × 10^−4^M

*Abs*_∞_ being the absorbance obtained at the end of the process, and k_3_ and “*n*” being the Avrami constant and exponent, respectively. Figure S22 shows how the second half of the Abs = f(t) obtained during the tyramine calibration study (Fig. 4) fit with (13) (*n* = 1, in all cases). Taking this into account, the full Abs = f(t) is given by the product of equations (7) and (13):

14 $$\mathrm{Abs}={\mathrm{K}}_{\mathrm{A}\mathrm{uPt}}\ \left(1-{\mathrm{e}}^{-{\mathrm{k}}_3\mathrm{t}}\right)\left(\frac{1-{\mathrm{e}}^{-{\mathrm{A}}_{\mathrm{A}\mathrm{uPt}}\mathrm{t}}}{{\mathrm{e}}^{-{\mathrm{A}}_{\mathrm{A}\mathrm{uPt}}\mathrm{t}}+{\mathrm{B}}_{\mathrm{A}\mathrm{uPt}}}\right)$$

A_AuPt_ and B_AuPt_ being described by similar equations as (8) and (9) respectively. However, *k*_1_ and *k*_2_ are different from *k*_1,Au_ and *k*_2,Au_, and K_AuPt_ groups the corresponding factors and the molar absorptivity.

Figure S23 shows that the Abs = f(t) representations obtained for different tyramine concentrations (used during the calibration study) fit very well with equation (14). Figure S24 shows how the A_AuPt_ linearly changes with the tyramine concentration, as expected according to (8), which allows the model to be validated; the same is valid for k_3_ (Figure S25), as expected from the Avrami model.

In summary, the Abs = f(t) is a combination of a classic nucleation/growth process with an aggregation of the formed NP.

IV) Analytical figures of merit

The results obtained during a calibration study under the optimum conditions are shown in Fig. 4 and S26. As can be seen, the blank signal is very low and the drift is partially avoided. The dynamic range depends on the analytical parameter chosen. A linear relationship is obtained using the area corresponding to the first 20 min (Figure S27), according to the following equation:

15 $${\mathrm{Area}}_{20\ \min }=0.4607\ \left[\mathrm{Tyramine}\right]+0.026\ {\mathrm{R}}^2=0.996$$

The linear relationship goes up to 250 μM, the quantification limit (LoQ) is 1 μM, the detection limit (LoD) is 0.3 μM, and the precision is 3.4% (*n* = 5, using 5 μM Tyramine). LoD was calculated using 3σ/m (where σ is the standard deviation of the blank and m is the slope of the calibration plot). However, if the absorbance at the end of the Abs = f(t) is used (60 min in Fig. 4), a second-order polynomial equation is obeyed from 0.5 μM up to 25 μM, so the LoQ is reduced. Moreover, if the absorbance at longer reaction times is used, higher sensitivity can be obtained, but also a higher blank signal. Since, these last parameters require longer measurement time, the Area_20_min_ is proposed.

These results show that the use of Au_Pt_ allows an improvement of the LoQ of at least 25 times compared with the use of Au(III) alone; this LoQ is in the order of that obtained using the classical TMB/HRP/H_2_O_2_ indicating-system and allows us to consider nanoparticle generation as a real analytical alternative.

E) Interference studies and application

The combination of the metals Au(III) and Pt(II) allows new achievements in interference control. Putrescine (Put), cadaverine (Cad), and histamine (His), the most common biogenic amines along with tyramine in food samples, were studied. Put and Cad alone give no signal, and a 2:1 Put(Cad) to tyramine ratio does not modify the tyramine signal (Fig S28A-B). Regarding His, the most important interference for tyramine determination, the results obtained indicate that histamine reduced the analytical signal due to tyramine, but to a lower extent (Fig S28C). So, while using Au(III) alone, the maximum His to tyramine ratio allowed (signal reduction about 15%) is 1:10; when Pt(II) is present, this ratio increases up to 2:1. This is probably due to the strength of the complexes that Pt(II) forms with histamine [21].

A real sample of cured cheese was studied where previously the tyramine concentration had been determined through the HRP:TMB method, finding a tyramine concentration of 2.7 ± 0.2 × 10^−4^ M (*n* = 3) (Fig.S29A). Due to the interferences previously mentioned, the sample was analyzed by the standard addition obtaining a similar concentration, 2.6 ± 0.3 × 10^−4^M (*n* = 3) (Fig. S29B).

F) Final considerations

This methodology presents several advantages compared to our previous studies where only Au(III) is employed, such as a lower LoD, a wider linear range, and less signal drift in the measurements. Nevertheless, low concentrations of tyramine require longer measurement times to obtain the appropriate signal. For this reason, as has been shown mathematically, the rate of formation of NPs is proportional to the concentration of tyramine. Another drawback is the sigmoidal response (discussed in previous studies [16]) which requires the use of second-degree equations, linearizations, or alternative parameters such as areas. Regarding the interferences of the method, although improvements are also obtained, it is still necessary to use the standard addition to avoid them.

Comparing this methodology with the classic colorimetric ones (such as HRP/TMB/H_2_O_2_), the advantages it offers in terms of avoiding lateral reactions have been previously commented. Nonetheless, it has also been verified that although longer measurement times are necessary, the LoQ reached is in the same order as with the classic methodologies. Finally, compared to those previously reported based on nanomaterials, it is important to highlight the low LOD and the simplicity of this method, since the use of pre-synthesized nanomaterials is not necessary. Table S1 summarizes the analytical figures of merit of the latest published methods for the determination of biogenic amines.

## Conclusions

The addition of Pt(II) allows both the generation of NP from Au(I) and the formation of bimetallic Au_Pt_NP. Considering the results given throughout this study, some conclusions can be obtained about the mechanisms of the NP formation:

1) From the Inverse method the most important conclusion is that Au(I) (and probably Pt(II)) seems to be able to replace O_2_ as a cofactor in the enzymatic reaction. Nevertheless, its analytical application is very limited.

2) In the Direct method, the product of the reaction (Tyr_Aldheyde_) is able to reduce metals. However, the enzyme of the reaction (TAO) (no other proteins) is necessary for the formation and stabilization of the NP.

This paper demonstrates that the use of bimetallic Au(III)/Pt(II) mixtures allows us to solve the most important problems found when only Au(III) is used in the formation of NPs during the enzymatic reaction, resulting in an improvement of the LOQ, the elimination of the signal drift and the smoothing of the His interference effect. It would be of interest to continue studying the use of other types of nanomaterials as colorimetric indicators for this type of methodology.

Moreover, the results suggest that it is worth studying other types of nanomaterials for later improvements in the analytical capabilities of this methodology competing with those based on the use of fluorophores or dyes.

## Supplementary information

ESM 1 (DOCX 2.33 mb)

## References

[1] Porstmann B, Porstmann T, Nugel E (1981). Comparison of chromogens for the determination of horseradish peroxidase as a marker in enzyme immunoassay. J Clin Chem Biochem.

[2] Osman AM, Wong KKY, Fernyhough A (2006). ABTS radical-driven oxidation of polyphenols: isolation and structural elucidation of covalent adducts. Biocehm Biophys Res Commun.

[3] Scott SL, Chen WJ, Bakac A, Espenson JH (1993). Spectroscopic parameters, electrode potentials, acid ionization constant, and electron exchange rates of the 2,2´-azinobis (3-ethylbenzothiazoline-6-sulfonate) radicals and ions. J Phys Chem.

[4] Navarro J, Sanz-Vicente I, Lozano R, de Marcos S, Galban J (2020). Analytical possibilities of putrescine and cadaverine enzymatic colorimetric determination in tuna based on diamine oxidase: A critical study of the use of ABTS. Talanta.

[5] Nasir M, Nawaz MH, Latif U, Yagub M, Hayat A, Rahim A (2017). An overview on enzyme-mimicking nanomaterials for use in electrochemical and optical assays. Microchim Acta.

[6] Diez-Buitrago B, Briz N, Liz-Marzan LM, Pavlov V (2018). Biosensing strategies based on enzymatic reactions and nanoparticles. Analyst.

[7] Tsogas GZ, Vlessidis AG, Giokas DL (2022). Analyte-mediated formation and growth of nanoparticles for the development of chemical sensors and biosensors. Microchim Acta.

[8] Navarro J, de Marcos S, Galban J (2020). Colorimetric-enzymatic determination of tyramine by generation of gold nanoparticles. Microchim Acta.

[9] Creighton JA, Eadon DG (1991). Ultraviolet-visible absorption spectra of the colloidal metallic elements. J Chem Soc Faraday Trans.

[10] Moodley KG, Nicol MJ (1977) Kinetics of reduction of gold(III) by platinum(II) and iron(III) in aqueous chloride solutions. J Chem Soc Dalton Trans (10):993. 10.1039/dt9770000993

[11] Peloso A (1975). Oxidation of platinum(II) complexes by tetrachloroaurate(III) ions in the presence of tetraethylamomnoium chloride. Coord Chem Rev.

[12] Jastrazb R, Lomozik L, Tylkowski B (2016). Complexes of biogenic amines in their role in living systems. Phys Sci Rev.

[13] Liu K, He Z, Curtin JF, Byrne HJ, Tian F (2019). A novel, rapid, seedless, in situsynthesis method of shape and size controllable gold nanoparticles using phosphates. Sci Rep.

[14] Zohar N, Chuntonov L, Haran G (2014). The simplest plasmonic molecules: metal nanoparticle dimers and trimers. J Photochem Photobiol C: Photochem Rev.

[15] He W, Zhou YT, Wamer WG, Hu X, Wu X, Zheng Z, Boudreau MD, Yin JJ (2013). Intrinsic catalytic activity of Au nanoparticles with respect to hydrogen peroxide decomposition and superoxide scavenging. Biomaterials.

[16] Camacho-Aguayo J, de Marcos S, Mora-Sanz V, Galban J (2022). Selective generation of gold nanostructures mediated by flavo-enzymes to develop optical biosensors. Biosens Bioelectron.

[17] Watzky MA, Finke RG (1997). Transition metal nanocluster formation kinetic and mechanistic studies. A new mechanism when hydrogen is the reductant: slow, continuous nucleation and fast autocatalytic growth. J Am Chem Soc.

[18] Finney EE, Finke RG (2008). Nanocluster nucleation and growth kinetic and mechanistic studies: a review emphasizing transition-metal nanoclusters. J Colloid Interface Sci.

[19] Jun YS, Zhu YG, Wang Y, Ghim D, Wu XH, Kim D, Jung H (2022) Classical and nonclassical nucleation and growth mechanisms for nanoparticle formation. Annu Rev Phys Chem 73(451). 10.1146/annurev-physchem-082720-100947 10.1146/annurev-physchem-082720-100947 35113740

[20] Avrami M (1941). Granulation, phase change and microstructure. Kinetic of phase change III. J. Chem. Phys..

[21] Oziminski WP, Garnuszek P, Bednarek E, Dobrowolski JC (2007). The platinum complexes with histamine: Pt(II)(Hist)Cl2, Pt(II)(Iodo-Hist)Cl2 and Pt(IV)(Hist)Cl2. Inorg Chim Acta.

