# Supplementary material for: In situ enzymatic generation of Au/Pt nanoparticles as an analytical photometric system: proof of concept determination of tyramine
Source: Mikrochim Acta. 2023 Mar 6;190(4):114. doi: 10.1007/s00604-023-05698-y (PMC9988730; doi:10.1007/s00604-023-05698-y)
Supplement: Supplementary file 1 — (DOCX 2.33 mb) [file 604_2023_5698_MOESM1_ESM.docx]

**ELECTRONIC SUPPLEMENTARY MATERIAL**

**In situ enzymatic generation of Au/Pt nanoparticles as an analytical photometric system: proof of concept determination of tyramine**

**Javier Camacho-Aguayo, Susana de Marcos, Carlos Felices and Javier Galbán^*^**

Nanosensors and Bioanalytical Systems (N&SB), Analytical Chemistry Department, Faculty of Sciences, Instituto de Nanociencia y Materiales de Aragón (INMA, Unizar-CSIC), University of Zaragoza, E50009 Zaragoza, Spain.

**SUMMARY**

**Section S1.- Au_Pd_ nanoparticles 2**

**A) Optimization**

Fig S1. Au (III) concentration effect 2

Fig S2. Pd (II) concentration effect 2

Fig S3. TAO concentration effect 3

Fig S4. pH and buffer concentration effect 3

**B) Characterization**

Fig. S5. TEM images and EDX spectra 4

**C) Analytical figures of merit**

Fig S6. Dynamic and linear range 5

Fig S7. Histamine interference 5

**Section S2 Au_Pt_ nanoparticles 6**

**A) Characterization**

Figure S8. Characterization of Au_Pt_ nanoparticles-Direct Method 6

Figure S9. Characterization of Au_Pt_ nanoparticles-Inverse method 6

**B) Inverse method**

Figure S10. Inverse method: Addition order 7

Figure S11. Inverse method: Addition time 7

Figure S12. Inverse method: Au (III): Pt (II) ratio 8

Figure S13. Inverse method: Calibration 8

Figure S14: Inverse method: Effect of the catalase 9

Figure S15: Inverse method: Linearity 9

**C) Direct method**

Figure S16. Direct method: Effect of Au (III): Pt (II) concentration 10

Figure S17. Direct method: Effect of the pH 12

Figure S18. Direct method: Effect of the TAO concentration 12

Figure S19. Direct method: Effect of the temperature 13

Figure S20. Direct method: Product and enzyme (TAO) role 13

Figure S21. Direct method: Peroxide effect 14

Figure S22. Direct method: Theoretical study I 14

Figure S23. Direct method: Theoretical study II 15

Figure S24. Direct method: Theoretical study III 15

Figure S25. Direct method: Theoretical study IV 16

Figure S26. Direct method: Calibration (Abs_60_) 16

Figure S27. Direct method: Calibration (Area) 16

Figure S28. Direct method: Interferences 17

Figure S29. Direct method: Real (cheese) sample 18

**Table 1 Comparison to other methods 18**

**Section S1.- Au_Pd_ NPs**

**A) Optimization**


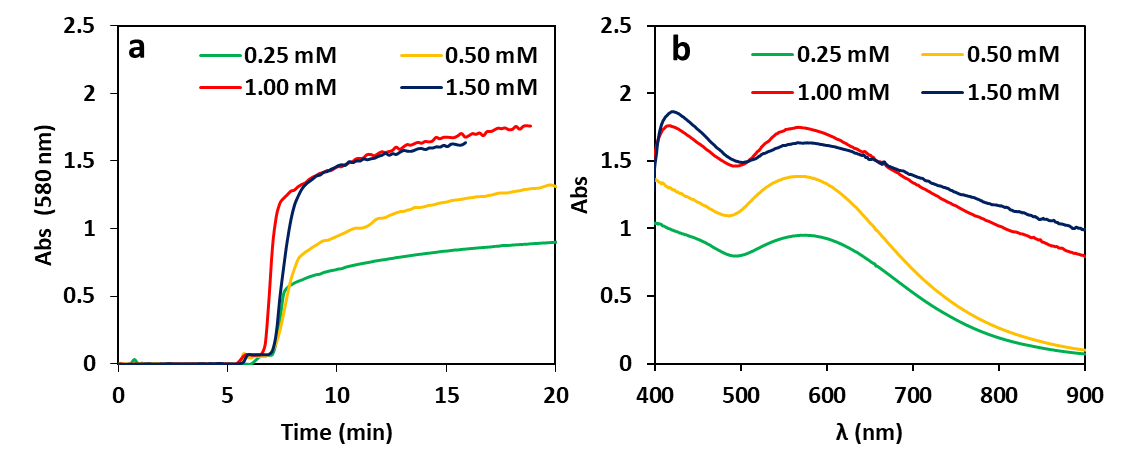


**Figure S1. Au (III) concentration effect on the Au_Pd_ nanoparticles generation.**

**a)** Variation of the absorbance (λ=580 nm) during the Au_Pd_NPs formation with the concentration of Au (III)

**b)** Spectra obtained at the end of the reaction.

**Comment:** It is observed the higher the [Au (III)] the higher the formation of Au_Pt_NPs but the wider the band spectra. Then, Au (III) 0.5 mM was chosen as the optimal.

**Experimental conditions:** [Pd (II)]=1.5 mM, [TAO]=0.5 U/mL; [Tyramine]=5·10^-4^ M; Phosphate Buffer 0.3 M pH 7; Tª=40ºC.


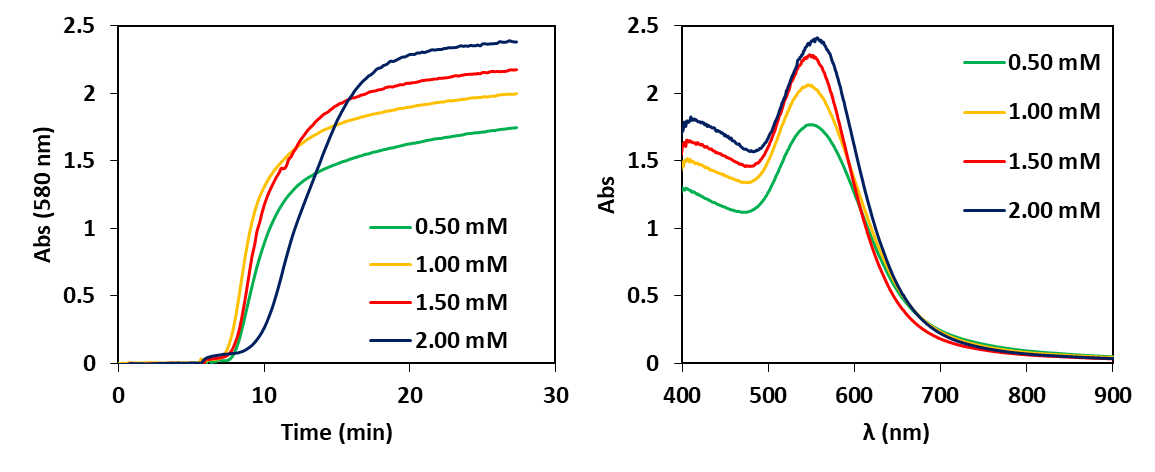


**Figure S2.- Pd(II) concentration effect.**

**a)** Variation of (λ=580 nm) during the Au_Pd_NPs formation with the concentration of Pd (II) and **b)** Spectra obtained at the end of the reaction

**Comment:** It is observed the higher the [Pd (III)] the higher the formation of Au_Pt_NPs but the slower the reaction rate. Then, Pd (II) 1.5 mM was chosen as the optimal.

**Experimental conditions:** [Au (III)]=0.5 mM; [TAO]=0.5 U/mL; [Tyramine]=5·10^-4^ M; Phosphate Buffer 0.3 M pH 7; Tª=40ºC.


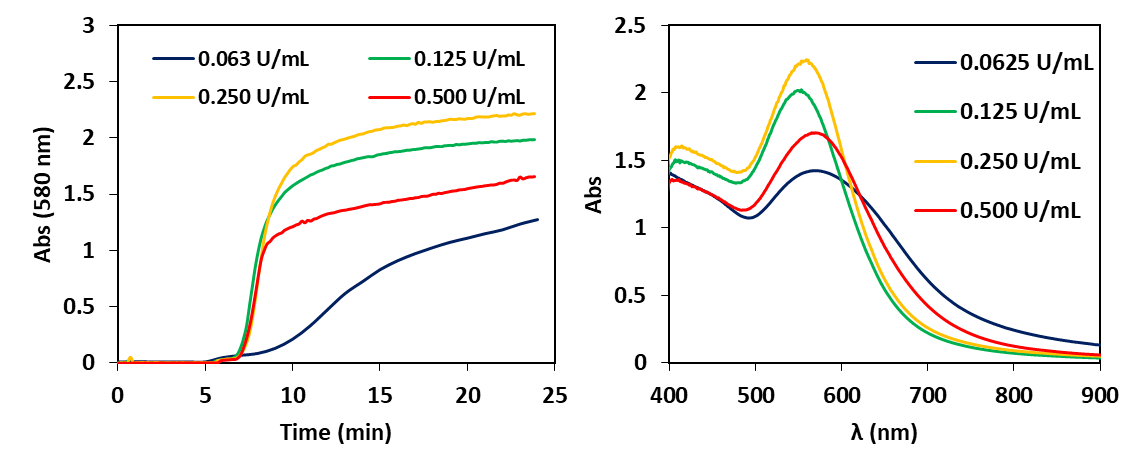


**Figure S3.- TAO concentration effect.**

**a)** Variation of (λ=580 nm) during the Au_Pd_NPs formation with the concentration of TAO and

**b)** Spectra obtained at the end of the reaction

**Comment:** It is observed that after a positive effect of TAO on the absorbance, the Au_Pt_NP formation rate decreases when the concentration of TAO is higher than 0.25 IU/mL. Then, 0.25 IU/mL was chosen as the optimal.

**Experimental Conditions:** [Au (III)]=0.5 mM; [Pd (II)]=1.5 mM; [Tyramine]=5·10^-4^ M; phosphate Buffer 0.3 M pH 7; Tª=40ºC.

**
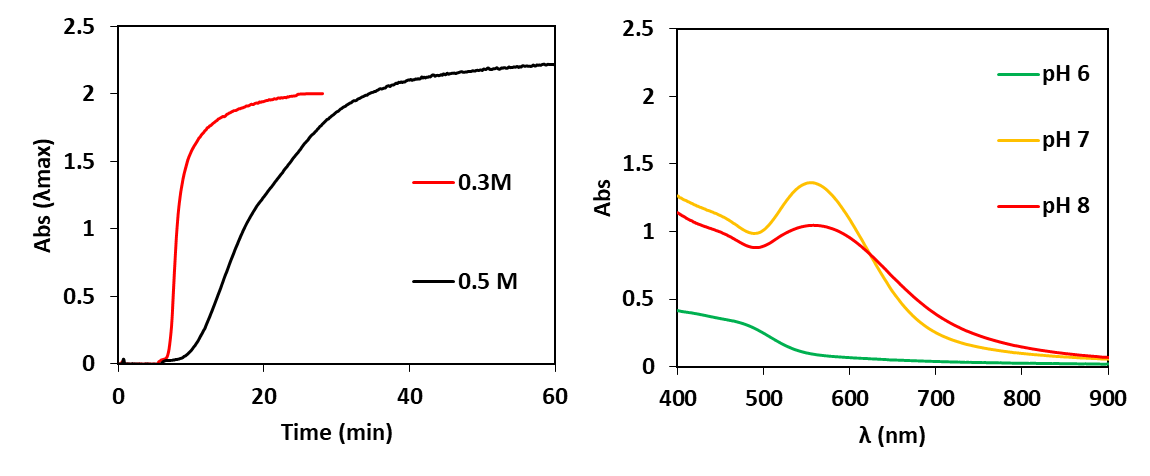
**

**Figure S4.- pH and buffer concentration effect.**

**a)** Variation of (λ=580 nm) during the Au_Pd_NPs formation with different phosphate buffer concentrations

**b)** Spectra obtained at the end of the reaction at different pH

**Comment:** Because of palladium salt is dissolved in HCl (6M), a phosphate buffer concentration higher than 0.2M is necessary. For that reason, buffer concentrations of 0.3 and 0.5 M are studied, observing that higher concentration slows down the reaction. This also explains why with pH 6 no NPs are formed. Hence a Phosphate Buffer pH 7 (0.3M) is chosen as optimal.

**Experimental Conditions:** [TAO]=0.25 U/mL; [Au (III)]=0.5 mM; [Pd (II)]=1.5 mM [Tyramine]=5·10^-4^ M; Tª=40ºC.

**B) Characterization**

**
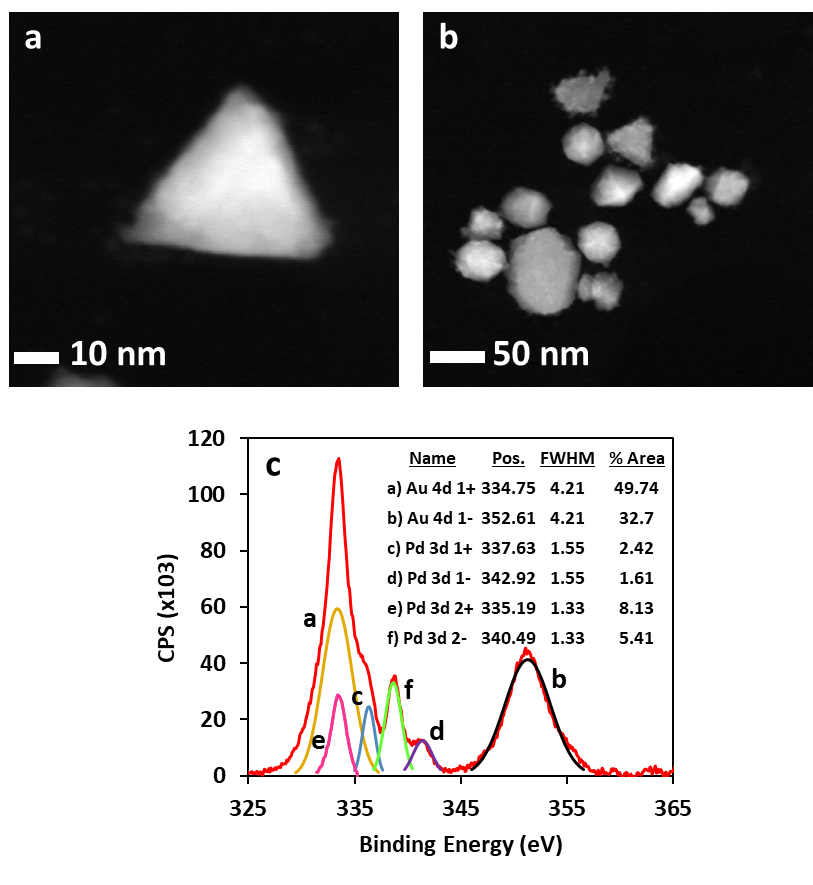
**

**Figure S5.- TEM images (a and b) and EDX spectra (c) of Au_Pd_ NPs.**

**C) Analytical figures of merits**

**A B**

**C**

**Figure S6. Dynamic and linear range.**

**Experimental Conditions:** [TAO]=0.25 U/mL; [Au (III)]=0.5 mM; [Pd (II)]=1.5 mM; phosphate Buffer 0.3 M pH 7; Tª=40ºC. Tyramine concentrations are indicated inside.

**A)** Abs=f(t) at 580 nm; **B)** Final spectra obtained for all the concentrations tested.; **C)** Final Absorbance at 580 nm

**Figure S7.- Study of the Histamine interference**

**Experimental Conditions:** [TAO]=0.25 U/mL; [Au (III)]=0.5 mM; [Pd (II)]=1.5 mM; [Tyramine]=1·10^-4^M; phosphate Buffer pH 7 (0.3M); Tª=40ºC.; Histamine concentrations are indicated inside.

**A)** Final spectra obtained for all the concentrations tested.; **B)** Final Absorbance at 580 nm

**Section S2.- Au_Pt_ NPs**

**A) Characterization**

*
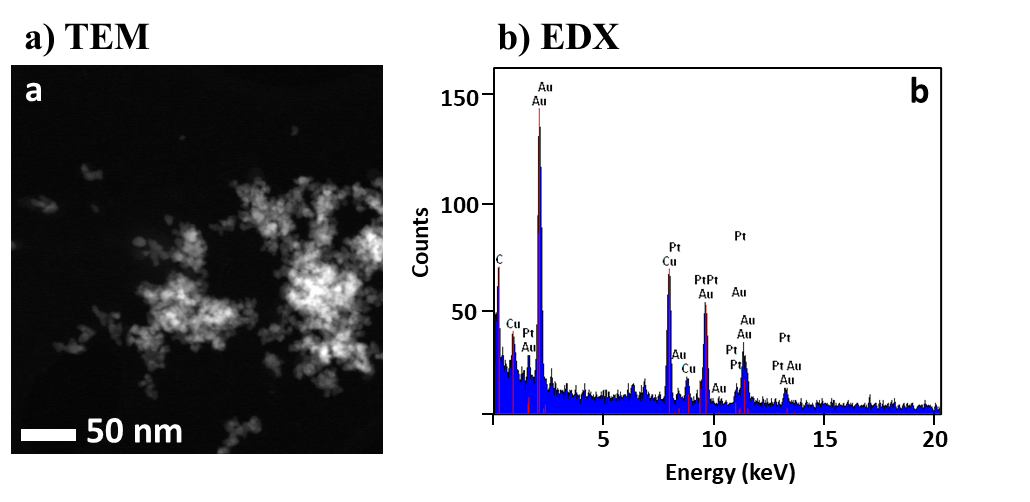
*

*
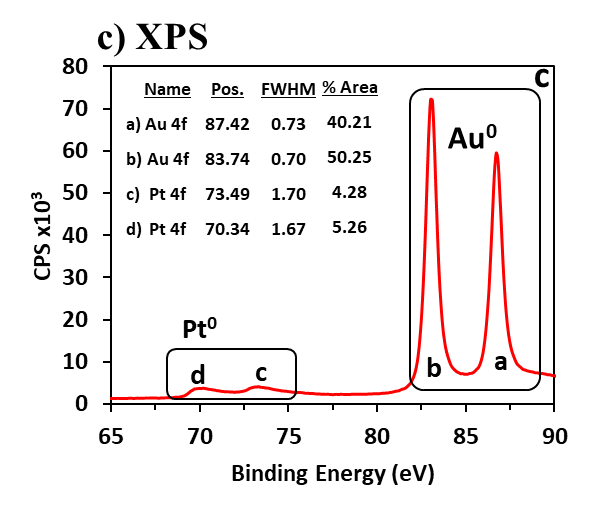
*

**Figure S8.- Characterization of Au_Pt_ NPs- Direct Method**

**a)** TEM**; b)** EDX**; c)** XPS

*
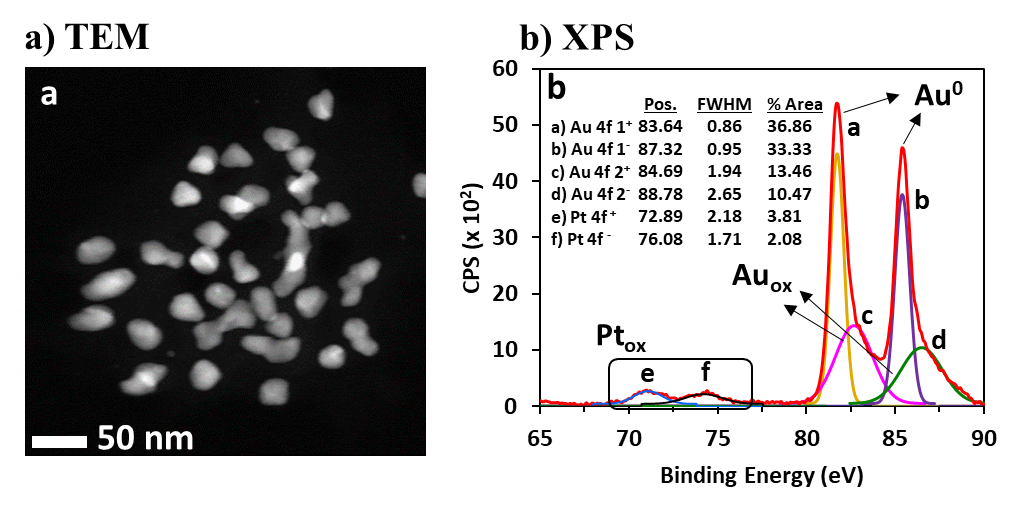
*

**Figure S9.- Characterization of Au_Pt_ NPs- Inverse Method**

**a)** TEM**; b)** XPS

**B) Inverse Method**


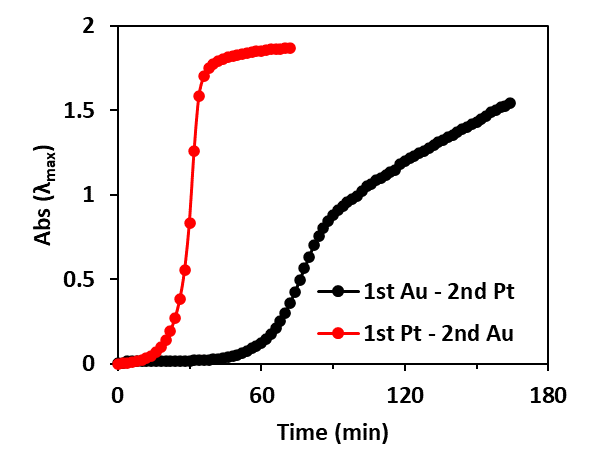


**Figure S10. Inverse method: Addition order**. Effect of the ion metals addition order on the Abs=f(t) representations obtained at 540 nm:

(**^__^**) Au (III) is added and then Pt (II);

(**^__^)** Pt (II) is added and then Au (III);

**Experimental Conditions:** [TAO]=0.5 U/mL; [Au (III)]=0.5 mM; [Pt (II)]=0.5 mM; [Tyramine]=1·10^-4^ M; phosphate buffer 0.1 M pH 7; Tª=40ºC.

**
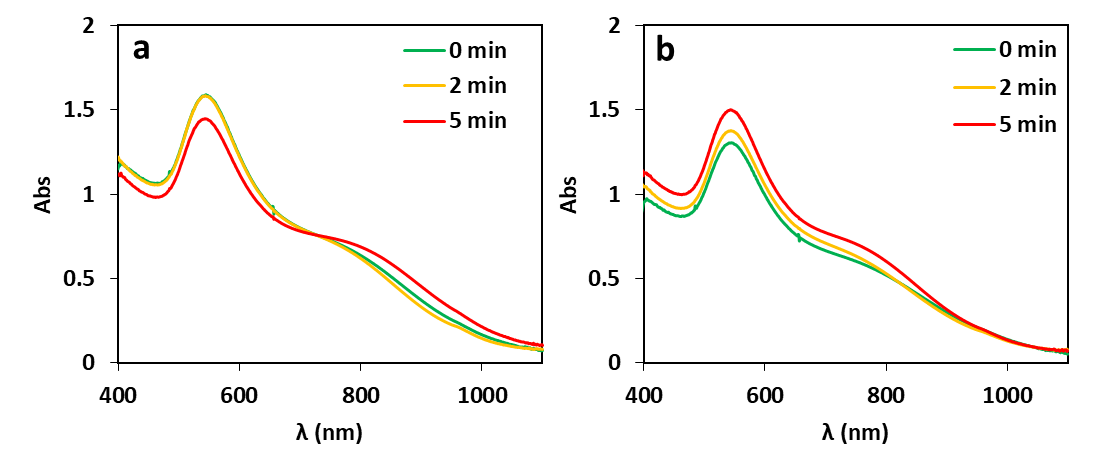
**

**Figure S11.- Inverse method: Addition time.** Absorption spectra obtained during the study of the addition time of the metals, according to the scheme given in Figure 2

**a)** Effect of t_1_ when t_2_=5 min; **b)** Effect of t_2_ when t_1_=5 min.

**Experimental Conditions:** [TAO]=0.5 U/mL; [Au (III)]=0.5 mM; [Pt (II)]=0.5 mM; [Tyramine]=5·10^-4^ M; phosphate buffer 0.1 M pH 7; Tª=40ºC.

**
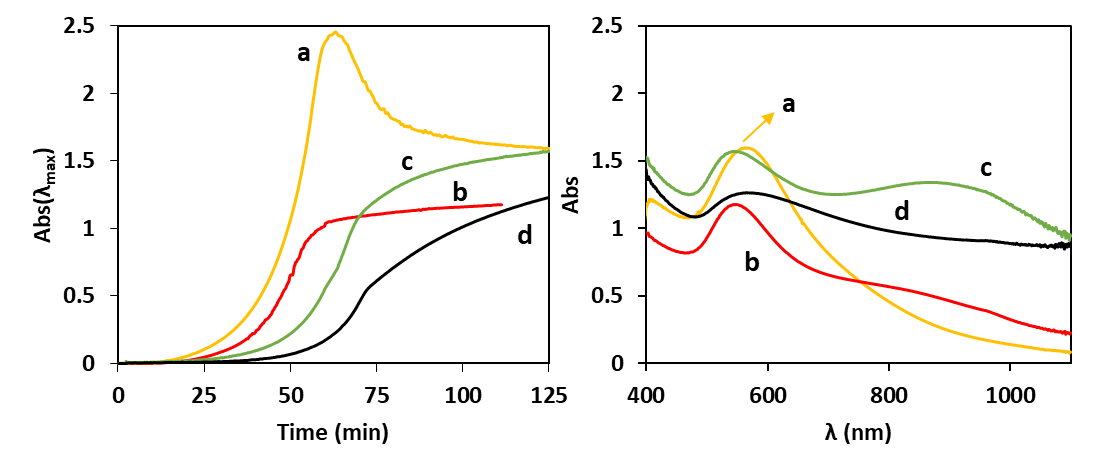
**

**B**

**A**

**Figure S12. Inverse method: Study of different Au (III):Pt(II) ratio.**

**A)** Variation of the absorbance (λ=540 nm) **B)** final spectra obtained

The following concentrations and ratio were tested:

**a)** [Au(III)]=1 mM; [Pt (II)]=1 mM; Ratio Pt/Au=1

**b)** [Au(III)]=0.5 mM; [Pt (II)]=0.5 mM; Ratio Pt/Au=1

**c)** [Au(III)]=0.5 mM; [Pt (II)]=1 mM; Ratio Pt/Au=2

**d)** [Au(III)]=0.5 mM; [Pt (II)]=2 m;; Ratio Pt/Au=4

**Experimental Conditions:** [TAO]=0.5 U/mL; [Tyramine]=1·10^-4^ M; phosphate buffer 0.1 M pH 7; Tª=40ºC.

**
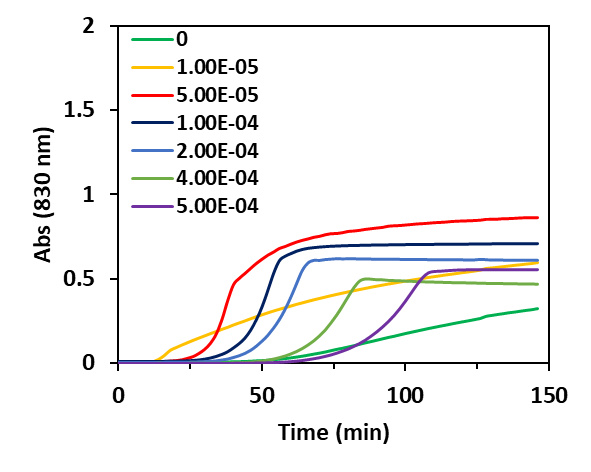
**

**Figure S13: Inverse method:** Calibration study using the inverse method

**Experimental Conditions:** λ=830 nm. [TAO]=0.5 U/mL; [Tyramine]=x M; [Au (III)]=0.5 mM; [Pt (II)]=0.5 mM; Phosphate buffer 0.1 M pH 7; Tª=40ºC.

**
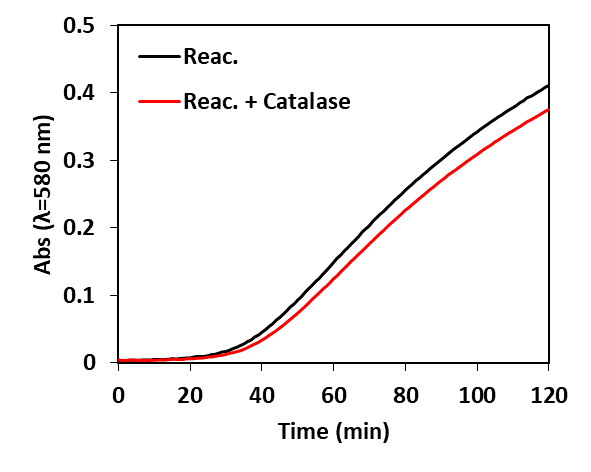
**

**Figure S14: Inverse method:** Variation of the absorbance (λ=540 nm) of the Au_Pt_NPs

**a)** in the absence, and **b)** in the presence, of Catalase (50 U/mL).

**Experimental Conditions:** [TAO]=0.5 U/mL; [Tyramine]=1·10^-4^ M; [Au (III)]=0.5 mM; [Pt (II)]=0.5 mM; Phosphate buffer 0.1 M pH 7; Tª=40ºC.

**Figure S15. Inverse method: Calibration study.** Variation of the (Abs_540_-Abs_830_ at 120 min) versus concentration of tyramine.

**Experimental Conditions:** [TAO]=0.5 U/mL; [Au (III)]=0.5 mM; [Pt (II)]=0.5 mM; Phosphate buffer 0.1 M pH 7; Tª=40ºC.

**C) Direct Method**


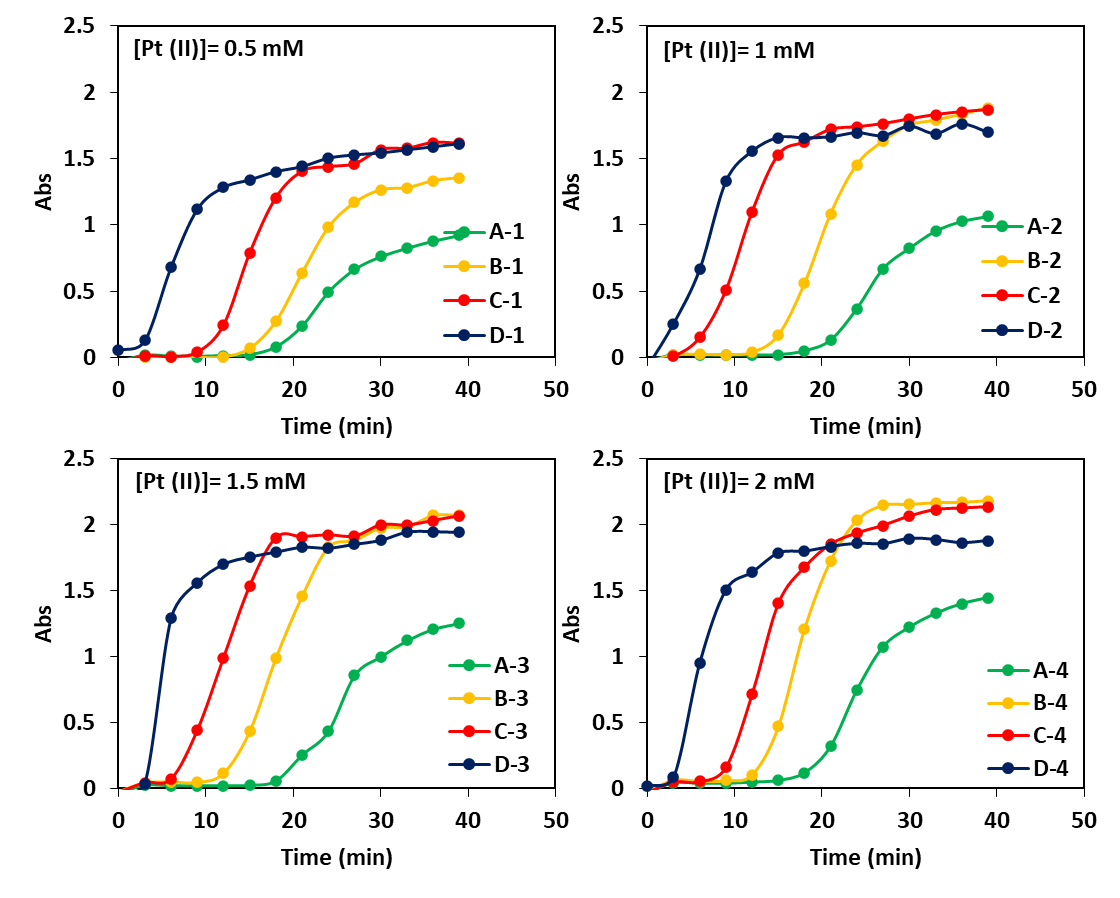


**Figure S16A. Direct method: Au (III): Pt (II) concentration.**

Variation of the absorbance (λ=580 nm) for different Au(III) and Pt(II) concentrations and ratios. In each figure Pt(II) concentrations is constant (as indicated) and Au(III) concentrations change according to the following:

**A:** [Au(III)]=0.25 mM.

[Pt(II)] = 0.5 mM (A-1), 1 mM(A-2), 1.5 mM (A-3), 2.0 mM (A-4)

**B:** [Au(III)]=0.50 mM.

[Pt(II)] = 0.5 mM (B-1), 1 mM(B-2), 1.5 mM (B-3), 2.0 mM (B-4)

**C:** [Au(III)]=1.0 mM.

[Pt(II)] = 0.5 mM (C-1), 1 mM(C-2), 1.5 mM (C-3), 2.0 mM (C-4)

**D:** [Au(III)]=1.5 mM.

[Pt(II)] = 0.5 mM (D-1), 1 mM(D-2), 1.5 mM (D-3), 2.0 mM (D-4)

**Experimental Conditions**: [TAO]=0.5 U/mL; [Tyramine]=1·10^-4^ M; phosphate buffer 0.1 M pH7 ; Tª=25ºC;

**
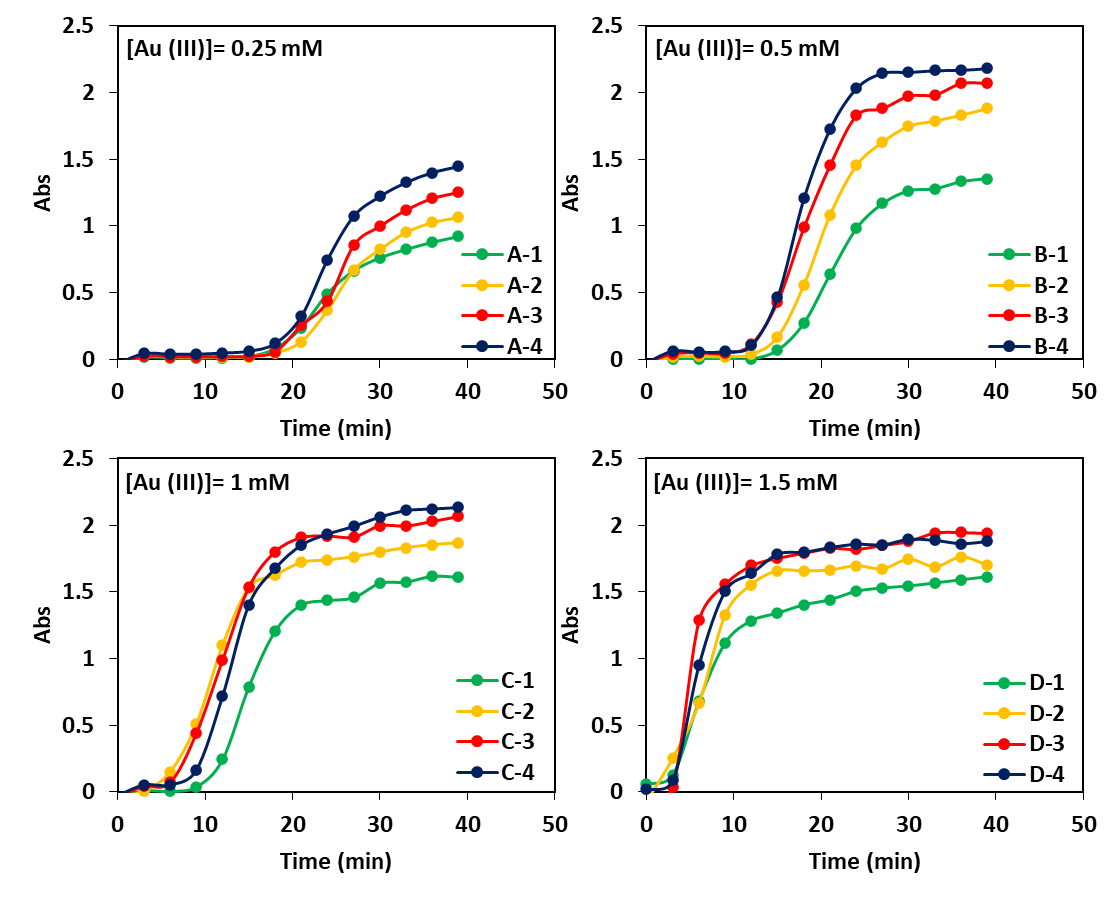
**

**Figure S16B. Direct method: Au (III): Pt (II) concentration.** Variation of the absorbance (λ=580 nm) for different Au(III) and Pt(II) concentrations and ratios.

In each figure Au(III) concentrations is constant and Pt (II) change according to the nomenclature used in Figure S16A.


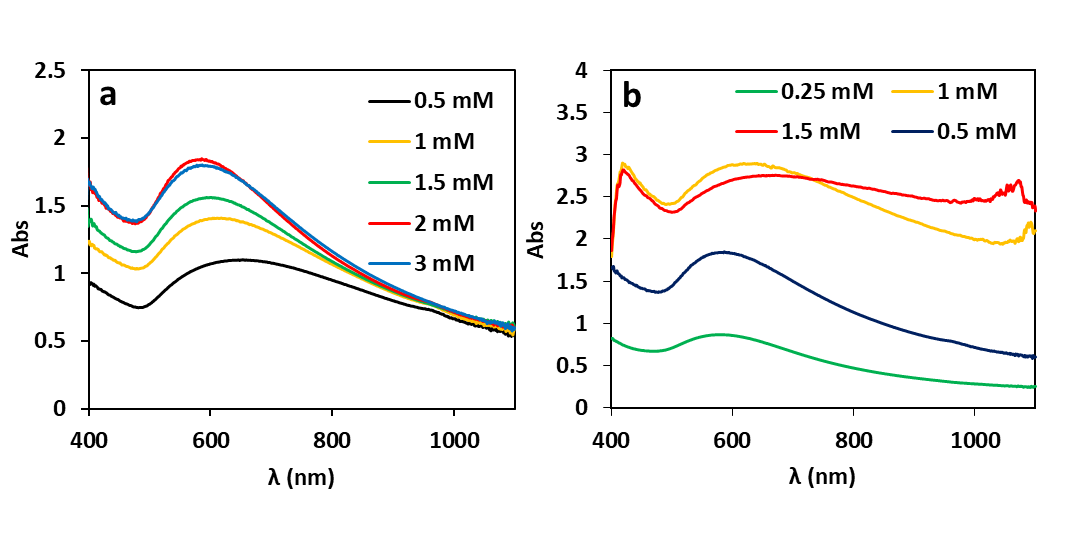


**S16C.-** Spectra of the Au_Pt_ NPs obtained with different concentrations of:

**a)** Pt (II) (as indicated inside figure)

**Experimental Conditions**: [Au (III)]=0.5 mM; [TAO]=0.5 U/mL; [Tyramine]=1·10^-4^ M; phosphate buffer 0.1 M pH 7 ; Tª=25ºC;

**b)** Au (III) (as indicated inside figure)

**Experimental Conditions**: [Pt (II)]=1.5 mM; [TAO]=0.5 U/mL; [Tyramine]=5·10^-4^ M; phosphate buffer 0.1 M pH 7 ; Tª=25ºC; [Pt (III)]= 1.5 and


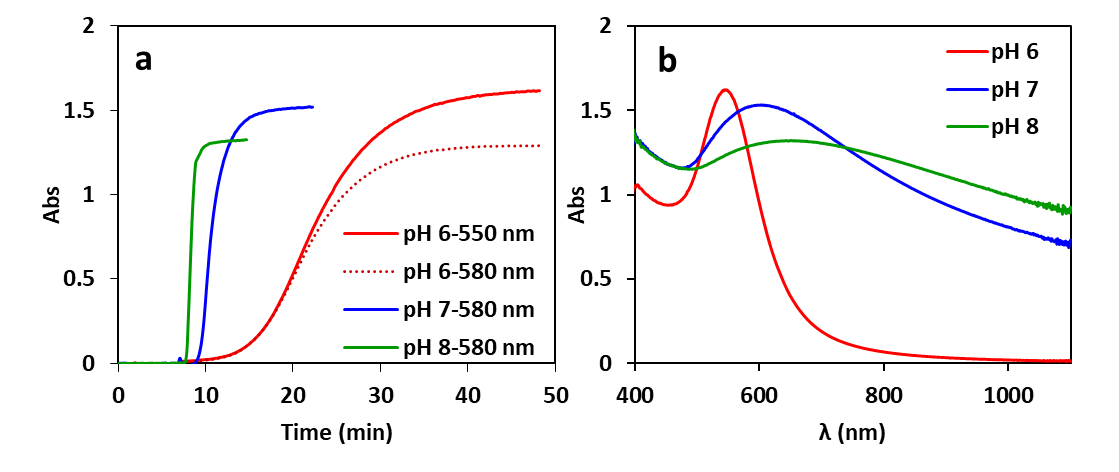


**Figure S17. Direct method: Effect of the pH**

**a)** Variation of the absorbance (λ=580 nm) during the formation of Au_Pt_NPs,

**b)** the final spectra obtained at different pH

**Experimental Conditions:** [TAO]=0.5 U/mL; [Au (III)]=0.5 mM; [Pt (II)]=1 mM; [Tyramine]=1·10^-4^ M; phosphate buffer 0.1M; Tª=25ºC.

**
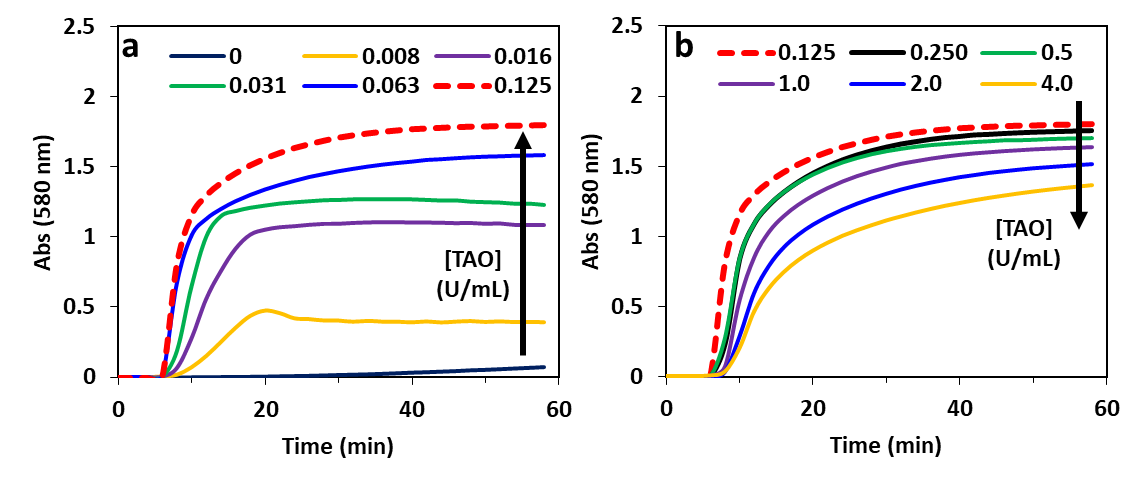
**

**
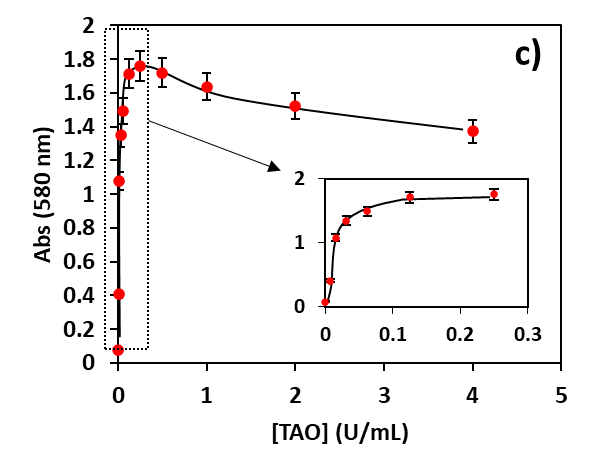
**

**Figure S18.- Direct method: Effect of TAO concentration.** Variation of the absorbance (λ=580 nm) during the formation of Au_Pt_NPs using different TAO concentrations. [Au (III)]=0.5 mM; [Pt (II)]=2 mM; [Tyramine]= 5·10^-5^ M; phosphate buffer 0.1 M pH 7. In all cases, TAO concentrations were as indicated in figures.

**a)** TAO concentrations below 0.125 U/mL; **b)** TAO concentrations above 0.125 U/mL.

**c)** Abs_60_min_ at 580 nm for different concentrations of TAO (U/mL);

**
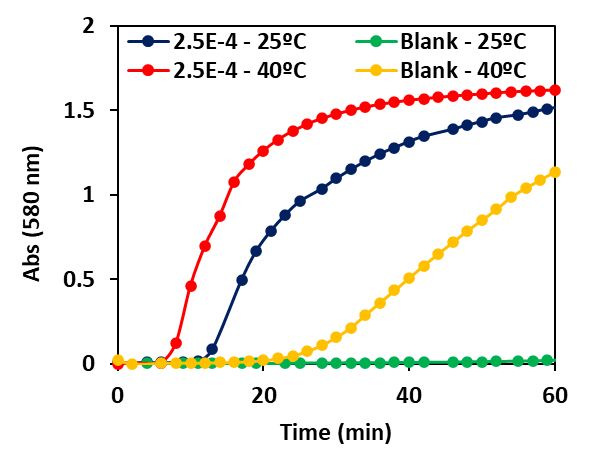
**

**Figure S19.- Direct method: Effect of the temperature.** Variation of the absorbance (λ=580 nm) during the formation of Au_Pt_NPs at different temperatures.

**Experimental Conditions:** [TAO]=0.5 U/mL; [Au (III)]=0.5 mM; [Pt (II)]=1 mM; [Tyramine]=5·10^-5^ M; phosphate buffer 0.1 M pH 7.


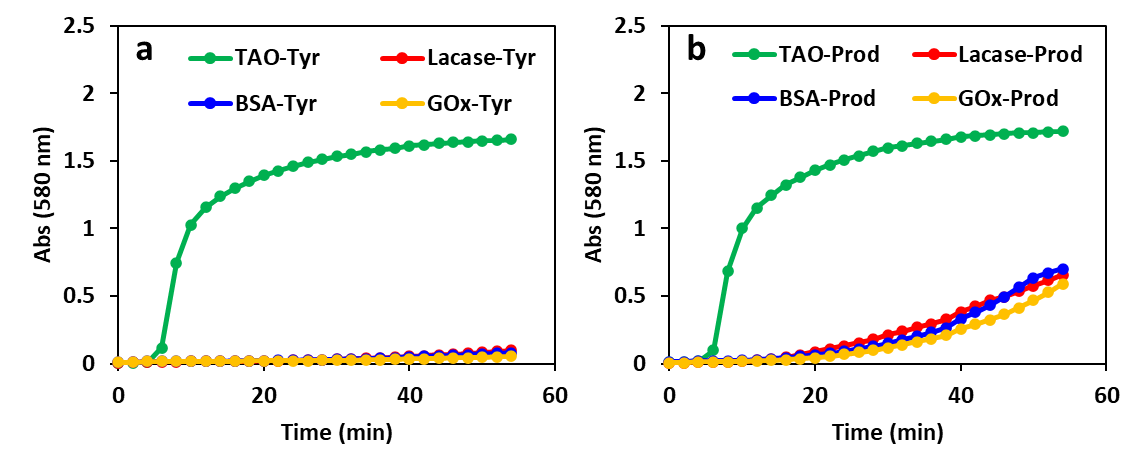


**Figure S20. Direct method: Product and enzyme (TAO) role.** Abs=f(t) (λ=580 nm) when

**a)** tyramine and **b)** product (Tyr_aldehyde_) are mixed with different enzymes or proteins.

**Comment:** Although in previous studies the product of the reaction was not able to fully explain the formation of the nanoparticles, it seems that in this new approach the product would play a much more important role, being the responsible of the nanoparticles formation. It is important also to highlight the stabilizing role of the enzyme (TAO), since no similar results were obtained with other enzymes and proteins.

**Experimental Conditions:** [TAO]=0.25 U/ mL=0.10 mg/mL; [Protein/Enzyme]=0.10 mg/mL; [Au (III)]=0.5 mM; [Pt (II)]=1 mM; [Tyramine]=[Product]=1·10^-4^ M; phosphate buffer 0.1 M pH 7; Tª=25ºC.


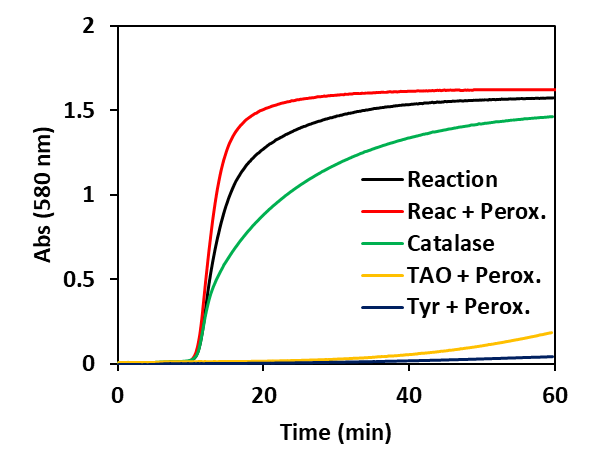


**Figure S21.- Direct method: Peroxide effect.** Variation of the absorbance (λ=580 nm) during the formation of Au_Pt_NPs at different situations where the peroxide (H_2_O_2_) can be involved. [Au (III)]=0.5 mM; [Pt (II)]=1 mM; phosphate buffer 0.1 M pH ; Tª=25ºC.

**Reaction**: [TAO]=0.5 U/mL; [Tyramine]=1·10^-4^ M.

**Reac + Perox**.: [TAO]=0.5 U/mL; [Tyramine]=1·10^-4^ M; [H_2_O_2_]=1·10^-4^ M.

**Reac + Catalase**: [TAO]=0.5 U/mL; [Catalase]=50 U/mL; [Tyramine]=1·10^-4^ M.

**TAO + Perox**: [TAO]=0.5 U/mL; [H_2_O_2_]=1·10^-4^ M.

**Tyr + Perox**: [Tyramine]=2.5·10^-4^ M; [H_2_O_2_]=1·10^-4^ M.

**Comment:** The role of H_2_O_2_ is still not clear in the formation of NPs, therefore some studies have indicated that H_2_O_2_ is able to growth previously formed NP and others studies highlight its etching role. In this approach, if the experiment is carried out in presence of catalase (*Reac + Catalase*), which allows to remove the H_2_O_2_ formed during the enzymatic reaction, the kinetic is affected, making the reaction slower. Otherwise, when more peroxide is added (*Reac + Perox*), it is seen how the formation of the nanoparticles accelerates. Nevertheless, peroxide is not able of generate NPs by itself (*TAO/Tyr + Perox*)


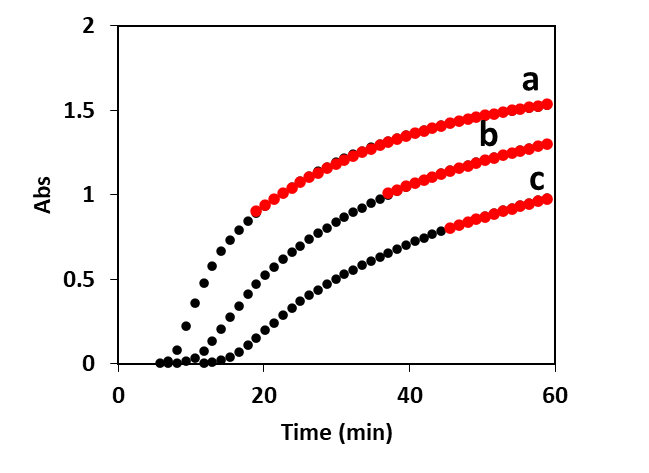


**Figure S22. Direct method: Theoretical study I:** Fitting of the second-half of Abs=f(t) obtained in the calibration line to the Avrami model. Black lines show the experimental absorbance values and red lines show the predicted absorbance at 580 nm values according to the model.

**a)** [Tyramine]= 2.5·10^-5^M. **b)** [Tyramine]= 1.5·10^-5^M. **c)** [Tyramine]= 1.0·10^-5^M.

**Experimental conditions:** [TAO]=0.25 U/mL=0.10 mg/mL; [Au (III)]=0.5 mM; [Pt (II)]=1 mM; phosphate buffer 0.1M (pH=7).

**
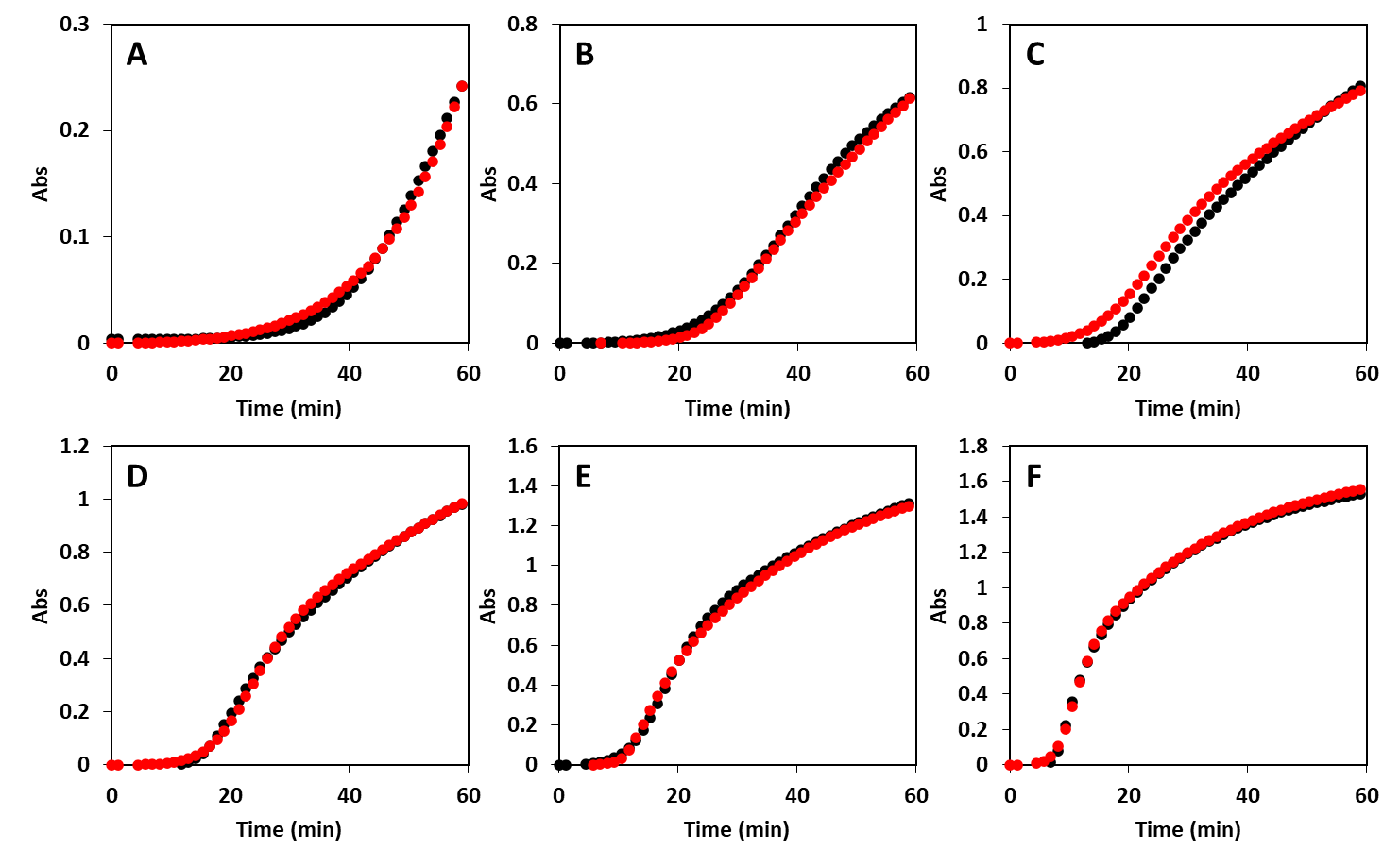
**

**Figure S23. Direct method: Theoretical study II.** Fitting of the Abs=f(t) obtained for different points of the calibration line to the kinetic model.

**Black circles =** experimental values obtained at 580 nm.

**Red circles =** the predicted absorbance values at 580 nm according to the model.

**A)** C_Tyr_= 2.5·10^-6^M . **B)** C_Tyr_= 5.0·10^-6^M . **C)** C_Tyr_= 7.5·10^-6^M .

**D)** C_Tyr_= 1.0·10^-5^M . **E)** C_Tyr_= 1.5·10^-5^M . **F)** C_Tyr_= 2.5·10^-5^M .

Experimental conditions: [TAO]=0.25 U/mL=0.10 mg/mL; [Au (III)]=0.5 mM; [Pt (II)]=1 mM; phosphate buffer 0.1M (pH=7).

**
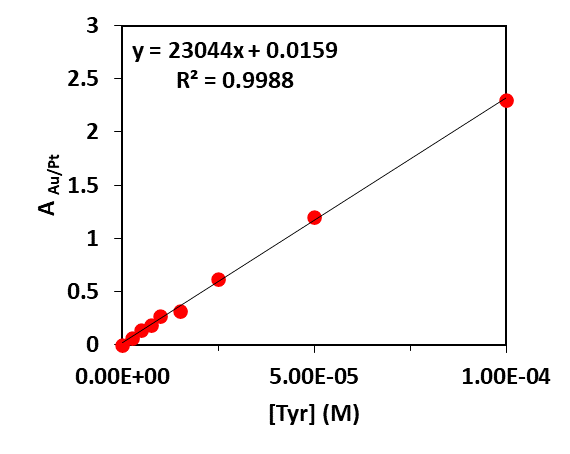
**

**Figure S24. Direct method: Theoretical study III.** A_Au/Pt_ values derived from the application of the model to the Abs=f(t) representations shown in figure 4 of the main manuscript.

**
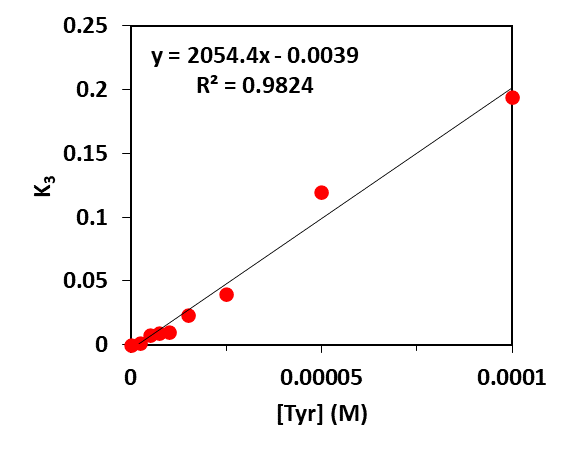
**

**Figure S25. Direct method: Theoretical study IV.** k_3_ values derived from the application of the model to the Abs=f(t) representations shown in figure 5.

**
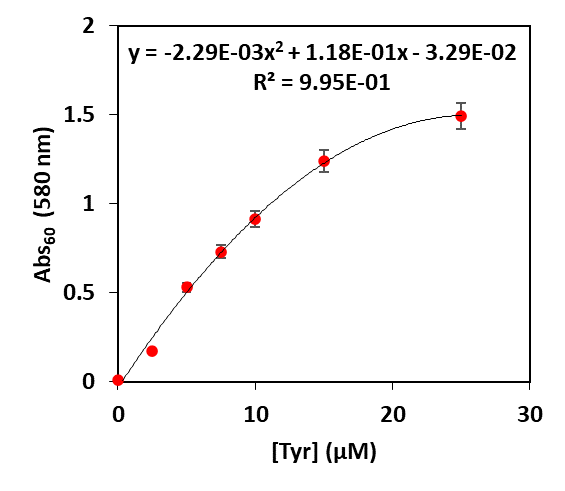
**

**a b**

**Figure S26. Direct method:** Calibration curve using the Abs_60_ of the Abs=f(t) representations (figure 5 of the main manuscript).**a)** Full range; **b)** Second degree range.

**
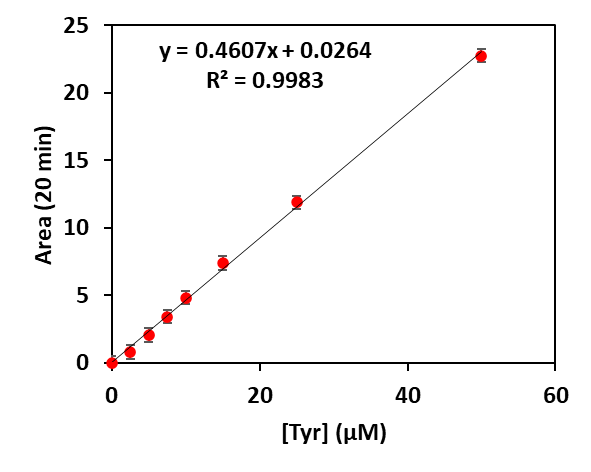
**

**Figure S27. Direct method:** Calibration curve using the area of the Abs=f(t) representations during the first 20 minutes of the formation of NPs.

1. **Putrescine**


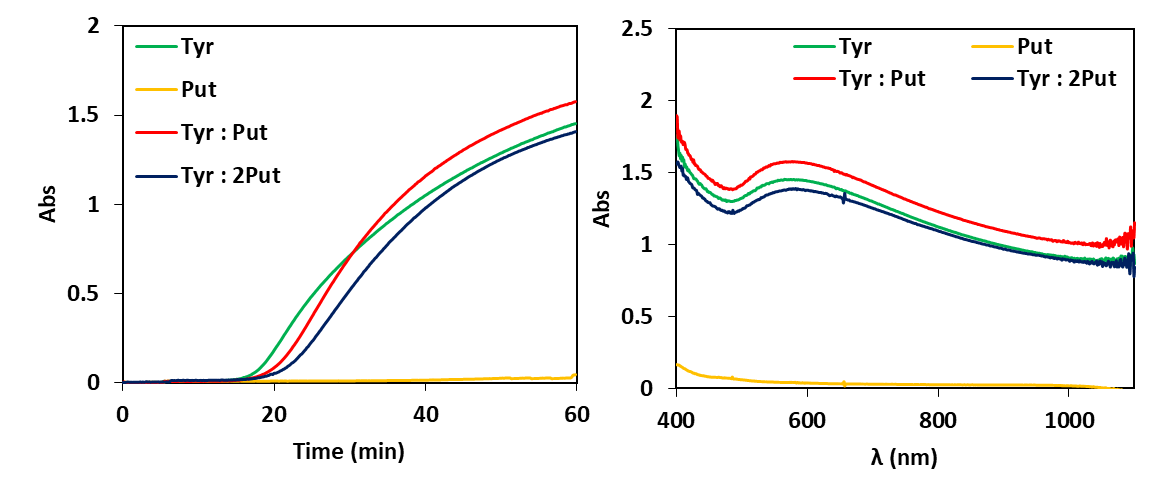


1. **Cadaverine**

**
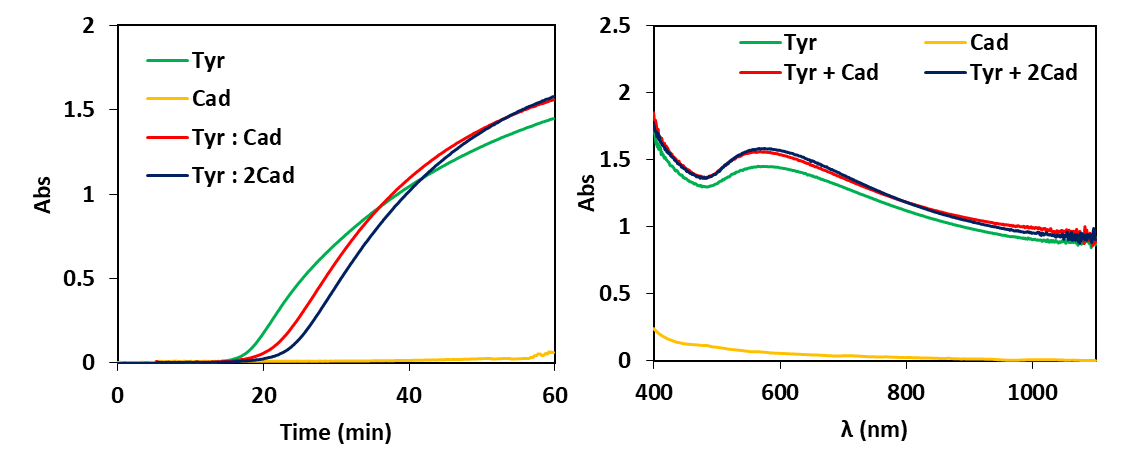
**

1. **Histamine**


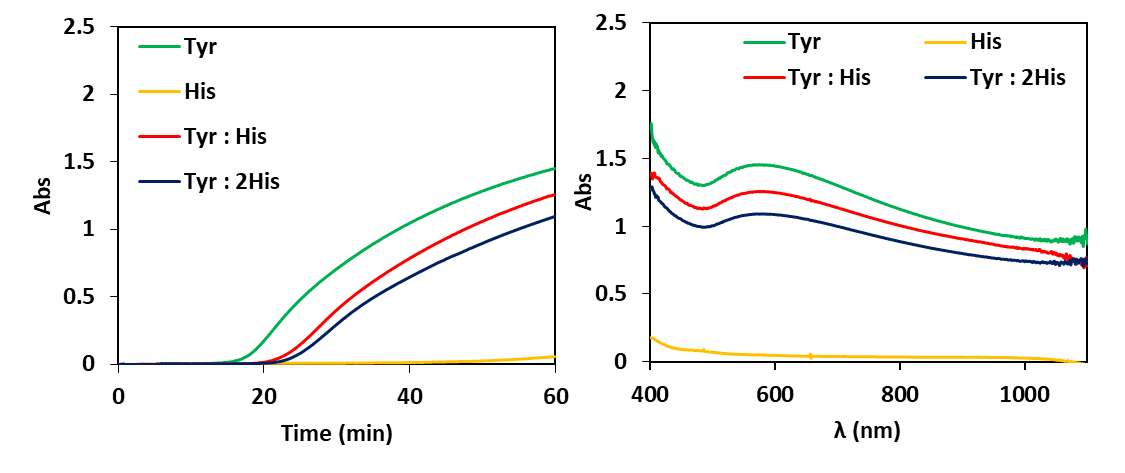


1. **Interference level expressed as % (Tyramine alone 100%)**


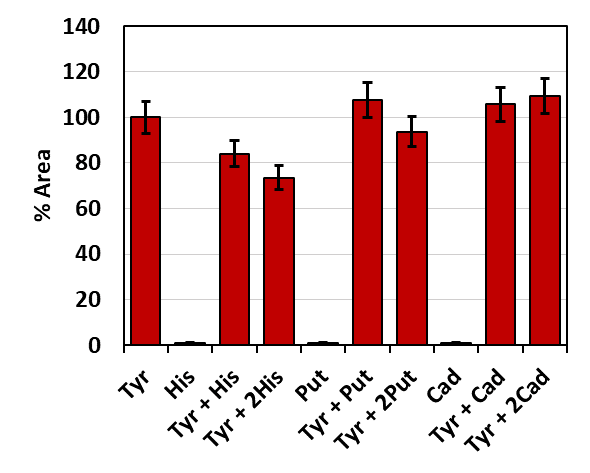


**Figure S28. Direct method: Interferences.** Abs=f(t) (λ=580 nm) obtained during the formation of Au_Pt_NPs, and the final spectra, obtained in the interference study.

**A)** Cadaverine **B)** Putrescine **C)** Histamine **D)** Signal expressed as % area Tyramine

**Experimental Conditions:** [TAO]=0.25 U/mL=0.10 mg/mL; [Au (III)]=0.5 mM; [Pt (II)]=1 mM; [Biogenic Amine]=1·10^-5^ M; phosphate buffer 0.1 M pH 7; Tª=25ºC.


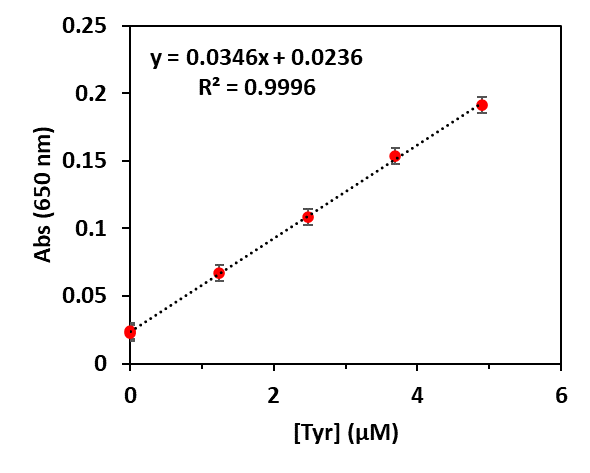

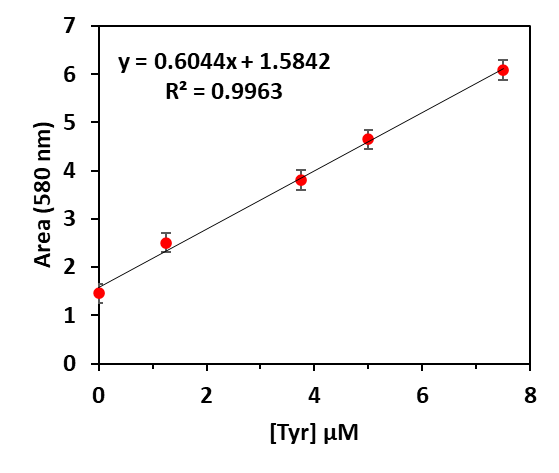


**A B**

**Figure S29:** **Tyramine determination in cheese**

**A)** TAO:HRP:TMB reference method and standard addition. **Absorbance** (λ=650 nm); phosphate buffer 0.1 M pH 7; [TAO]=0.5 U/mL; [HRP]=0.12 U/mL; [TMB]= 2·10^-4^ M.

**B)** Au_Pt_NPs absorbance values obtained at 580 nm; application of the standard addition method. **Experimental conditions:** [TAO]=0.25 U/mL; [Au (III)]=0.5 mM; [Pt (II)]=1 mM; phosphate buffer 0.1 M pH 7; Tª=25ºC.

**Table S1**

| **Composition of sensor** | **Response time** | **Detected signal** | **Analytical**  **Range** | **LOD** | **Ref** |
| --- | --- | --- | --- | --- | --- |
| AgNPs (preliminary HPTLC separation of the analyte) | - | Raman  (λ_exc_:633 nm) | 30-80 mg/kg | - | 1 |
| Combination of the pH indicator dye (2-fluoro-4-[4-(2-hydroxyethanesulfonyl)-phenylazo]-6-methoxyphenol (GJM-492)) and Remazol Brilliant blue R inmovilized of cellulose microplates | 1.5 h | CIE lab color space | 0.3-30 mg/kg | - | 2 |
| Luminiscence readout cellulose acetate nanofibers embedded with Py-1 | 20 min | Fluorescence with RGB/digital camera | 1.37-13.7 mg/kg | 0.4 mg/kg | 3 |
| Microtiter plate with sensor film based on Py-1 embebed in Hypan HN80 | 10 min | Fluorescence/Fluostar  Optima microtiter plate reader | 0.5 - 70.0 mg/kg  (histam.) | 0.165  mg/kg  (histam.) | 4 |
| Gra-QDs@MIPs | 50 min | Fluorescence enhancement | 0.07 – 12 mg/kg | 0.02 mg/kg | 5 |
| Melanin-UCNPs  (NaGdF_4_:Yb/Er@ NaYF_4_) | 45 min | Fluorescence quenching | 0.02 – 4.57 mg/kg | 0.004  mg/kg | 6 |
| Fluorescent organic nanoparticles (FONs) with tetrapodal receptor | - | Fluorescence  Spectrum  changes | 27.4 – 219.5 mg/kg | 0.05 mg/kg | 7 |
| Immunoassay based on enzyme-enabled growth of AuNStarts | 45 min | Color generation | 0.31 – 20 mg/kg | 0.25 mg/kg | 8 |
| Immunoassay combined with magnetic separation and UCNP | 30 min  + treat. | Fluorescence enhancement | 0.5-100 μg/kg | 0.1 μg/kg | 9 |
| Flu@Eu-dbia  (Lanthanide-MOF) | 2 min | Fluorescence increase | 0.03-9.59 mg/kg | 0.01 mg/kg | 10 |
| CD-MIP  (Test-strips) | - | Fluorescence quenching | 0.5-10 mg/kg | 0.059 mg/kg | 11 |
| Polyamine Carbon Quantum dots (PA@CQDs) | 15 min | Fluorescence enhancement | 5 - 400  μg/kg | 0.55 μg/kg | 12 |
| AuNPs formation | ≈ 30 min | Color generation | 3.4-45.3 mg/kg | 0.46 mg/kg | 13 |
| This work | Time ([Tyr]) | Color generation | 0.14 – 6.85 mg/kg | 0.04 mg/kg | * |

1. *Wang L, Xu XM, Chen YS, Ren J, Liu YT. HPTLC-FLD-SERS as a facile and reliable screening tool: exemplarily shown with tyramine in cheese. J Food Drug Anal. 2018;26:688–95.*
2. *Schaude, C., Meindl, C., Fröhlich, E., Attard, J., & Mohr, G. J. (2017). Developing a sensor layer for the optical detection of amines during food spoilage. Talanta, 170, 481-487.*
3. *Yurova NS, Danchuk A, Mobarez SN, Wongkaew N, Rusanova TY, Baeumner AJ, et al. Functional electrospun nanofibers for multimodal sensitive quantitation of biogenic amines in food via a simple dipstick assay. Anal Bioanal Chem. 2018;410:1111–21.*
4. *Khairy GM, Azab HA, El-Korashy SA, Steiner MS, Duerkop A. Validation of a fluorescence sensor microtiterplate for biogenic amines in meat and cheese. J Fluoresc. 2016;26:1905–16.*
5. *Wang, Q., & Zhang, D. (2018). A novel fluorescence sensing method based on quantum dot-graphene and a molecular imprinting technique for the detection of tyramine in rice wine. Analytical Methods, 10(31), 3884-3889.*
6. *Wang, H., Lu, Y., Wang, L., & Chen, H. (2019). Detection of tyramine and tyrosinase activity using red region emission NaGdF4: Yb, Er@ NaYF4 upconversion nanoparticles. Talanta, 197, 558-566.*
7. *Kaur, N., Kaur, M., Chopra, S., Singh, J., Kuwar, A., & Singh, N. (2018). Fe (III) conjugated fluorescent organic nanoparticles for ratiometric detection of tyramine in aqueous medium: A novel method to determine food quality. Food chemistry, 245, 1257-1261.*
8. *Luo, L., Luo, S. Z., Jia, B. Z., Zhang, W. F., Wang, H., Wei, X. Q., ... & Yang, J. Y. (2022). A high-resolution colorimetric immunoassay for tyramine detection based on enzyme-enabled growth of gold nanostar coupled with smartphone readout. Food Chemistry, 396, 133729.*
9. *Zhang, B., Sheng, W., Liu, Y., Huang, N., Zhang, W., & Wang, S. (2020). Multiplexed fluorescence immunoassay combined with magnetic separation using upconversion nanoparticles as multicolor labels for the simultaneous detection of tyramine and histamine in food samples. Analytica Chimica Acta, 1130, 117-125.*
10. *Ji, C., Zhang, J., Fan, R., Chen, Y., Zhang, Y., Sun, T., & Yang, Y. (2023). A lanthanide-MOF based host–guest intelligent dual-stimulus response platform for naked-eye and ratiometric fluorescence monitoring of food freshness. Journal of Materials Chemistry C.*
11. *Qiao, D., Zhang, Z., Wang, L., Sheng, W., Deng, Q., & Wang, S. (2021). In-situ preparation of molecularly imprinted fluorescent sensing test strips for on-site detection of tyramine in vinegar. Microchemical Journal, 160, 105638.*
12. *Salman, B. I., Hassan, Y. F., Eltoukhi, W. E., & Saraya, R. E. (2022). Quantification of tyramine in different types of food using novel green synthesis of ficus carica quantum dots as fluorescent probe. Luminescence, 37(8), 1259-1266.*
13. *Navarro, J., de Marcos, S., & Galbán, J. (2020). Colorimetric-enzymatic determination of tyramine by generation of gold nanoparticles. Microchimica Acta, 187(3), 1-8.*
